# Depression and Suicidal Ideation Among Men who have sex with men and Transgender Women in Chiang Mai, Thailand

**DOI:** 10.12688/f1000research.181753.2

**Published:** 2026-07-10

**Authors:** Kyaw Sein Lynn, Linda Aurpibul, Nuntisa Chotirosniramit, Natthapol Kosashunhanan, Kriengkrai Srithanaviboonchai, Awirut Oon-arom

**Affiliations:** 1 School of Health Sciences Research, Research Institute for Health Sciences, Chiang Mai University, Chiang Mai, Chiang Mai, 50200, Thailand; 2 Research Institute for Health Sciences, Chiang Mai University, Chiangmai, CHIANG MAI, 50200, Thailand; 3 Psychiatry, Chiang Mai University Faculty of Medicine, Chiang Mai, Chiang Mai, 50200, Thailand

**Keywords:** Depression, Suicidal Ideation, PrEP users, men who have sex with men, transgender women

## Abstract

**Background:**

High burden of depression and suicidality were reported among men who have sex with men (MSM) and transgender women (TGW) globally. Currently, in Thailand, many of them can access to pre-exposure prophylaxis (PrEP) services for HIV prevention. We determined depression severity and suicidal ideation in PrEP users and non-users and associated factors.

**Methods:**

A cross-sectional study was conducted at two HIV prevention clinics in Chiang Mai, Thailand. Inclusion criteria were: 1) aged ≥20 years, 2) having sexual behaviours consistent with MSM or TGW; and 3) being a current PrEP user (using PrEP for ≥6 months) or non-user (never used PrEP). Socio-demographic data was collected by face-to-face interview. Depression and suicidal ideation were assessed using the Patient Health Questionnaire-9 (PHQ-9), and the Columbia-Suicide Severity Rating Scale (C-SSRS), respectively. Group differences between PrEP users and non-users were examined and multiple linear regression was applied to identify associated factors. Given the low prevalence of suicidal ideation (5.3%), a univariable logistic regression was conducted as an exploratory analysis.

**Results:**

A total of 150 MSM and TGW (75 PrEP users; 75 non-users) were included. Clinically significant depression (PHQ-9 scores ≥10) was observed in four PrEP users (5.3%) and 10 non-users (13.3%). PrEP users had significantly lower mean depression scores than non-users (3.80 ± 3.46 vs. 5.96 ± 3.76, *p* < 0.001). Suicidal ideation was uncommon, without difference between groups ( *p* = 0.47). In the adjusted model, frequency of current stress (B = 2.165, β = 0.352, p < .001) and PrEP use (B = −1.556, β = −0.216, p = .008) were independently associated with depression severity. PrEP use was not significantly associated with suicidal ideation in the exploratory analysis (OR = 1.714, 95% CI: 0.395–7.446, p = .472).

**Conclusions:**

PrEP use was associated with lower depression severity among MSM and TGW in Chiang Mai after adjustment for potential confounders, and frequency of current stress was the strongest associated factor. However, the cross-sectional design precludes causal conclusions, and the association between PrEP use and depression may reflect differences in healthcare engagement or social support rather than a direct effect of PrEP. These findings support integrating routine mental health screening and stress assessment into community-based HIV prevention programs serving MSM and TGW.

## Introduction

Sexual and gender diversity populations, including men who have sex with men (MSM), transgender women (TGW), and other Lesbian, Gay, Bisexual, Transgender, Queer or Questioning, and other sexual and gender minority identities (LGBTQ+ groups), experience a substantially higher burden of mental health problems than the general population. A recent systematic review and meta-analysis estimated that the pooled global prevalence of depression among MSM is about 35%, which is roughly three times more common than in the general male population ( Nouri et al., 2022) International research consistently shows that MSM and transgender women experience substantially higher levels of depression and suicidal ideation compared with the general population, with studies from the United States and India highlighting especially elevated risk among these groups ( Tomori et al., 2016; Wilton et al., 2018). Across diverse cultural contexts, stigma, discrimination, and social exclusion remain consistent drivers of poor mental-health outcomes among sexual and gender diversity populations ( Operario et al., 2022; Pellicane & Ciesla, 2022).

In Thailand, emerging evidence indicates that LGBTQ+ populations continue to experience substantial mental-health challenges. Recent studies have highlighted high levels of psychological distress among sexual and gender minority individuals, with a survey of Thai medical students reporting notably elevated rates of depressive and anxiety symptoms among those identifying as LGBTQ+ ( Vadhanavikkit et al., 2025). Research among TGW in Bangkok has also documented a high prevalence of depressive symptoms, with low perceived social support identified as a significant contributing factor ( Tantirattanakulchai & Hounnaklang, 2021). These findings reflect broader patterns observed across Southeast Asia, where stigma, discrimination, and social marginalization continue to shape mental-health disparities among MSM and TGW. Despite these concerns, systematic data on mental-health outcomes among key populations in northern Thailand remain limited, underscoring the need for focused research in this context.

Pre-exposure prophylaxis (PrEP) has become a central component of HIV prevention for MSM and TGW, offering highly effective protection when taken consistently ( Davies et al., 2016; Naswa & Marfatia, 2011; Turner et al., 2018). Beyond its biomedical benefits, the relationship between PrEP use and mental health is complex. Mental health concerns such as depression and anxiety are common among individuals at heightened risk for HIV and may hinder both PrEP uptake and adherence ( Hennessy et al., 2023; Miller et al., 2021). At the same time, PrEP-related stigma—arising from misconceptions within and outside LGBTQ+ communities—can influence willingness to initiate PrEP and contribute to psychological distress. Conversely, engagement with PrEP services may provide opportunities for social support and improved emotional well-being through regular contact with affirming healthcare settings. These intersecting dynamics highlight the importance of understanding mental-health profiles among PrEP users and non-users in Thailand ( Protiere et al., 2023; Sarah M. Wood, 2021; Sun et al., 2019; Yigit et al., 2022). In Thailand, PrEP delivery has expanded through the Key Population-Led Health Services (KPLHS) model, including in Chiang Mai, where community-based organizations provide HIV prevention services tailored to MSM, TGW, and other key populations. These settings aim to increase accessibility and reduce stigma related to HIV prevention. Although KPLHS clinics often offer more affirming environments compared with conventional healthcare facilities, stigma and limited service uptake remain challenges in several contexts.

However, existing Thai research has focused largely on PrEP-related stigma, provider attitudes, and implementation issues ( Chautrakarn et al., 2022; Vannakit et al., 2020), with limited attention to mental-health profiles of clients who used or did not use PrEP. Therefore, this study aimed to assess depression and suicidal ideation in MSM and TGW who were attending HIV prevention services in Chiang Mai, Thailand. We also looked for differences in mental health outcomes between PrEP users and non-users and identified potential associated factors. Understanding these patterns may support the development of integrated HIV prevention and mental-health services tailored to the needs of key populations.

## Materials and methods

### Study setting and participants

Since 2016, PrEP has been firstly implemented in Thailand and become a part of the HIV prevention package a few years later ( Phanuphak et al., 2018). Any hospital in the country can offer PrEP for those at risk for HIV acquisition, including the key populations. This cross-sectional study was conducted in Chiang Mai, Northern Thailand in 2025. At the time of this study, PrEP services are available at more than 20 government hospitals, more than 10 private hospitals, and several community-led clinics in Chiang Mai city also provide PrEP as a part of the campaign to minimize new HIV infection and achieve the HIV/AIDS elimination goal ( Data Center, Chiang Mai Provincial Health office, 2025). Among these, two are the principal PrEP service providers for MSM and TGW in Chiang Mai City: PIMAN Clinic, a research-based clinic located in the main Chiang Mai University campus, and MPLUS Polyclinic, which is a Key Population-Led Health Service (KPLHS) operated by a community-based organization. Together, these two clinics, which are 7 kilometres apart, serve the majority of MSM and TGW PrEP clients in Chiang Mai (approximately 850 active clients) and represent the two dominant service delivery models in Thailand—Healthcare facility and community-led. They were therefore selected purposively to maximize relevance, capture heterogeneity in service models, and ensure feasibility of participant recruitment. To align with programmatic definition of PrEP services target, the population of interest were biological males or transgender women who reported having sexual attraction and sexual activities with males.

Recruitment was conducted through a purposive non-probability sampling strategy, aiming for a total sample size of 150 participants. A sample size of 150 participants was selected based on the commonly reported depression prevalence of approximately 35% among MSM ( Nouri et al., 2022). With this sample, the 95% confidence interval around a 35% prevalence estimate would have a margin of error of about ±8%, which was considered adequate for descriptive purposes. Thus, the final sample size was deemed sufficient to address the study’s analytical aims and to explore associations between PrEP use, depression and suicidality among MSM and TGW in Chiang Mai. The study recruitment occurred from March to May 2025, with 75 participants enrolled from each clinic. Within each site, both MSM and TGW were included, ensuring balanced representation across service models. Inclusion criteria were: (1) aged ≥20 years; (2) having sexual behaviours consistent with MSM or TGW; and (3) either a current PrEP user (≥6 months) or a non-user (never used PrEP). Recruitment was conducted by trained research assistants, who reviewed clinic appointment logs, approached eligible clients in waiting areas, obtained written informed consent, and administered structured questionnaires in private consultation rooms. The specific variables and measures used were as follows:

### Data collection

• Socio-demographic Factors

Participant data included age, self-identified gender identity (categorized as male, female, transgender woman, non-binary), education level, living arrangement, employment status and monthly income. Some participants self-identified as female or non-binary on the gender item; however, they were included because they met the study’s behavioural eligibility criteria for MSM or TGW. This classification approach is consistent with programmatic definitions used in Thailand’s PrEP services, which prioritize behavioural risk over identity labels. Behavioural classification was prioritized over gender label to ensure accurate inclusion of key populations relevant to HIV prevention services. Community safety was assessed with the item: “Do you feel safe in your current living community?” Participants selected one of two response options: Always safe or not always safe. Stress level was measured with a single-item indicator: “How would you rate your current stress level?” Responses were dichotomized into High stress and Low/no stress based on participants’ self-evaluation. LGBTQ+ community involvement was evaluated with the question: “Are you actively involved in LGBTQ+ related community activities or groups?” with binary response options: Yes or No.

• Depression

Depression severity was measured using the Patient Health Questionnaire-9 (PHQ-9) ( Kroenke et al., 2001). This is a 9-item self-report scale assessing the frequency of depressive symptoms over the past two weeks on a 4-point scale (0 = ‘Not at all’ to 3 = ‘Nearly every day’). A total score was calculated by summing the responses, ranging from 0 to 27. The continuous PHQ-9 total score (‘Depression Score’) was categorized into ‘Depression Severity’ levels (Non-Minimal, Mild, Moderate, Moderately Severe, Severe) based on standard clinical cut-offs (0–4, 5–9, 10–14, 15–19, 20–27), and clinically significant depression was defined as PHQ-9 ≥ 10.The Thai version of the PHQ-9 has demonstrated good reliability and validity for screening major depression, with a Cronbach’s alpha of 0.79 yielding 84% sensitivity and 77% specificity ( Lotrakul et al., 2008).

• Suicidal Ideation/Risk

Suicidal ideation and risk were assessed using the Columbia-Suicide Severity Rating Scale (C-SSRS) Screen Version. ( Posner et al., 2011) It demonstrated good convergent and divergent validity with high sensitivity and specificity. The Thai version is also validated and accessible from the Columbia Lighthouse Project Website. ( The Columbia Lighthouse Project, 2016) Individual Yes/No responses to items covering wished dead, thoughts of killing self, method, intent, plan, and behaviour (Items 1–6) were collected. These responses were summarized into a single multi-categorical variable (‘Suicidal Thoughts’) representing different levels of suicidal ideation and risk.

### Data analysis

All data were entered and analyzed using the Statistical Package for the Social Sciences (SPSS), version 25. Data cleaning included verifying entries, checking for out-of-range values, and correcting entry errors.

Descriptive analyses were used to summarize sociodemographic, psychosocial, and outcome variables, presented as frequencies, percentages, means, and standard deviations. Group comparisons between PrEP users and non-users were conducted using χ ^2^ tests for categorical variables and independent-samples *t* tests (or Mann–Whitney U tests for non-normal distributions) for continuous variables.

Regression analyses were conducted to identify predictors of key outcomes. Multivariable linear regression models were used for the continuous outcomes of depression severity (PHQ-9 total score) and a binary logistic regression model was used for suicidal ideation. Covariates entered into all multivariable models included age, education, employment status, monthly income, community safety, stress level, social support, PrEP use, and LGBTQ+ community involvement.

For suicidal ideation, given the low prevalence in the sample (n = 8, 5.3%), a univariable binary logistic regression was conducted with PrEP use as the sole predictor. This approach was chosen in accordance with the standard guideline of requiring a minimum of 10 outcome events per predictor variable for stable regression estimates. This analysis is therefore presented as exploratory only, and findings should be interpreted with caution.

All reported findings therefore represent adjusted associations. Model assumptions were examined: residuals were inspected for linearity and homoscedasticity, and variance inflation factors (VIFs <2.0) indicated no problematic multicollinearity.

## Results

### Participant characteristics

A total of 150 participants were included in the analysis, comprising 75 PrEP users and 75 non-users. Sociodemographic and psychosocial characteristics by PrEP use status are presented in Table 1. Of the 150 participants, the majority self-identified as MSM (76.0%), followed by transgender women (16.0%), non-binary individuals (5.3%), and a small proportion biological males who identified themselves as female (2.7%).

**Table 1.** Comparison of Sociodemographic and Psychosocial Characteristics by PrEP Use Status

Characteristics	PrEP Users (n = 75) n (%)	Non-Users (n = 75) n (%)	Total (N = 150) n (%)	p-value
**Age group (years)**				**0.03**
20–40 years	65 (86.7%)	73 (97.3%)	138 (92.0%)	
After 40 years	10 (13.3%)	2 (2.7%)	12 (8.0%)	
**Education level**				**0.54**
High/Vocational school or lower	13 (17.3%)	**17 (22.7%)**	30 (20.0%)	
Undergraduate or higher	62 (82.7%)	58 (77.3%)	120 (80.0%)	
**Living arrangement**				**0.25**
Living alone	39 (52.0%)	31 (41.3%)	70 (46.7%)	
Living with others (family/friends)	36 (48.0%)	44 (58.7%)	80 (53.3%)	
**Employment status**				**0.02**
Unemployed/student	13 (17.3%)	26 (34.7%)	39 (26.0%)	
Employed	62 (82.7%)	49 (65.3%)	111 (74.0%)	
**Monthly income**				**1.00**
Low income (<20,000 THB or no income)	48 (64.0%)	49 (65.3%)	97 (64.7%)	
Higher income (>20,000 THB)	27 (36.0%)	26 (34.7%)	53 (35.3%)	
**Experience of discrimination**				**0.50**
Experienced discrimination	47 (62.7%)	42 (56.0%)	89 (59.3%)	
No discrimination	28 (37.3%)	33 (44.0%)	61 (40.7%)	
**Family/Society pressure**				**0.14**
High pressure	16 (21.3%)	25 (33.3%)	41 (27.3%)	
Low/No pressure	59 (78.7%)	50 (66.7%)	109 (72.7%)	
**Community safety**				**0.04**
Always safe	48 (64.0%)	35 (46.7%)	83 (55.3%)	
Not always safe	27 (36.0%)	40 (53.3%)	67 (44.7%)	
**Current stress level**				**0.01**
Low stress	75 (100.0%)	68 (90.7%)	143 (95.3%)	
High stress	0 (0.0%)	7 (9.3%)	7 (4.7%)	
**Social support**				**0.12**
Has support	75 (100.0%)	71 (94.7%)	146 (97.3%)	
No support	0 (0.0%)	4 (5.3%)	4 (2.7%)	
**LGBTQ+ Community involvement**				**<0.01**
Yes	66 (88.0%)	52 (69.3%)	118 (78.7%)	
No	9 (12.0%)	23 (30.7%)	32 (21.3%)	
**Total**	**75 (100%)**	**75 (100%)**	**150 (100%)**	

PrEP: pre-exposure prophylaxis; THB: Thai baht; LGBTQ: Lesbian, gay, bisexual, transgender, queers plus.

Significant differences between the two groups were observed for age group (p = 0.03), employment status (p = 0.02), community safety (p = 0.04), current stress level (p = 0.01), and LGBTQ+ community involvement (p < 0.01). Compared with non-users, PrEP users were more likely to be aged over 40 years (13.3% vs. 2.7%), employed (82.7% vs. 65.3%), report always feeling safe in their community (64.0% vs. 46.7%), and be involved in LGBTQ+ community activities (88.0% vs. 69.3%). Additionally, all PrEP users reported low stress levels, whereas 9.3% of non-users reported high stress. No significant differences were found regarding education level, living arrangement, monthly income, experience of discrimination, family/society pressure, or social support (all p > 0.05).

### Depression and suicidal ideation

Mental-health outcomes by PrEP use status are summarized in Table 2. The mean depression score was significantly lower among PrEP users (mean = 3.80, SD = 3.46) compared with non-users (mean = 5.96, SD = 3.76), with a mean difference of 2.16 (95% CI: 0.99–3.33; *p* < 0.001). Clinically significant depression (PHQ-9 ≥ 10) was observed in 4 PrEP users (5.3%) and 10 non-users (13.3%), indicating a higher proportion of moderate-to-severe depressive symptoms among those not using PrEP.

**Table 2.** Mental Health Outcomes Among PrEP Users and Non-Users

Variable	Those who use PrEP (mean, SD)	Those who do not use PrEP (Mean, SD)	Mean Difference (95% CI)	p-value
Depression Score	3.80, 3.46	5.96, 3.76	2.16 (0.99, 3.33)	<0.001
Suicidal Ideation	0.07, 0.25	0.04, 0.19	−0.027 (-0.10, 0.05)	0.47

PrEP: pre-exposure prophylaxis; SD: standard deviation; CI: confident interval

The frequencies of suicidal ideation were 7% among PrEP users and 4% among non-users, without difference between group.

### Factors associated with depression

A multiple linear regression model was conducted to examine predictors of depression severity ( Table 3) The results showed that frequency of current stress (B = 2.165, β = 0.352, p < .001) and PrEP use (B = −1.556, β = −0.216, p = .008) were significantly associated with depression severity. Higher stress frequency was associated with higher depression scores, whereas PrEP users had significantly lower depression scores compared with non-users after adjusting for age, education level, employment status, perceived community safety, and LGBTQ+ community involvement. No significant associations were observed for age, education level, employment status, perceived community safety, or LGBTQ+ community involvement.

**Table 3.** Linear Regression for Depression Severity

Predictors	B	SE	β (Beta)	t	p-value	95% CI for B	VIF
(Constant)	−0.849	2.363	—	−0.359	.720	[−5.521, 3.823]	—
Age in years	−0.030	0.039	−0.061	−0.761	.448	[−0.107, 0.047]	1.191
Education level	0.411	0.303	0.102	1.359	.176	[−0.187, 1.009]	1.062
**Frequency of current stress**	2.165	0.470	0.352	4.602	**<.001**	[1.236, 3.094]	1.093
**PrEP Use**	−1.556	0.581	−0.216	−2.680	**.008**	[−2.704, −0.408]	1.216
Employment status	0.106	0.198	0.043	0.537	.592	[−0.285, 0.497]	1.196
Perceived community safety	0.286	0.400	0.056	0.714	.476	[−0.505, 1.077]	1.150
LGBTQ+ community involvement	0.493	0.400	0.093	1.232	.220	[−0.298, 1.284]	1.072

PrEP: pre-exposure prophylaxis; SE: standard error; VIF: Variance Inflation Factor

PrEP use was independently negatively associated with lower depression scores (B = −0.231, p = 0.025) after adjusting for covariates. Other covariates, including age and PrEP adherence, were found as insignificant predictors. No multicollinearity concerns were identified (all VIF values <2).

### Factors associated with suicidal ideation

An exploratory univariable binary logistic regression was conducted to assess the association between PrEP use and suicidal ideation ( Table 4). PrEP use was not significantly associated with suicidal ideation (OR = 1.714, 95% CI: 0.395–7.446, p = .472). Although the estimated odds of suicidal ideation were higher among PrEP users than non-users, the wide confidence interval and lack of statistical significance indicate insufficient evidence of an association in this sample.

**Table 4.** Univariable binary logistic regression for suicidal ideation (Exploratory).

Predictor	B	SE	Wald	df	p value	OR (Exp B)	95% CI for OR
PrEP Use (Binary)	0.539	0.749	0.517	1	.472	1.714	[0.395, 7.446]
Constant	−3.178	0.589	29.088	1	<.001	0.042	—

## Discussion

This study examined associations between PrEP use, depression severity, and suicidal ideation among MSM and TGW in Chiang Mai, and identified sociodemographic and psychosocial factors associated with these outcomes. PrEP use was associated with lower depression scores, while suicidal ideation was uncommon and did not differ significantly by PrEP use status. In multivariable analyses, PrEP use and current stress remained independently associated with depression severity after adjustment for potential confounders. However, given the cross-sectional design, these findings should not be interpreted as evidence of causality.

The association between PrEP use and lower depression scores remained after adjustment for measured baseline differences, but unmeasured factors may still explain this relationship. Individuals who initiate and maintain PrEP may have greater healthcare engagement, higher health literacy, and stronger connections to community-based organisations, all of which may contribute to better mental health. Participation in PrEP programs may also provide access to social and peer support resources not assessed in this study. In addition, reverse causation is possible, whereby individuals with better psychological well-being are more likely to engage in PrEP care. Therefore, the observed association may reflect a combination of healthcare engagement, social support, and baseline mental health rather than a direct effect of PrEP itself. Longitudinal studies are needed to clarify these relationships.

Frequency of current stress was the strongest factor associated with depression severity in this sample (β = 0.352, p < .001), regardless of PrEP use status. This finding is consistent with evidence on the role of chronic psychosocial stress in driving mental health disparities among sexual and gender minority populations and underscores the importance of addressing stress as a modifiable target within HIV prevention services, independent of any PrEP-specific effects ( Kaprinis & Charalampakis, 2025; Meyer, 2003).

This study has several limitations. First, its cross-sectional design precludes causal inference and does not establish the temporal relationship between PrEP use and depression; therefore, the observed association should be interpreted cautiously. Second, participants were recruited from two community-based sites in a single province, which may limit generalizability to MSM and TGW in other settings or those not engaged in services. Third, self-reported measures of depression and suicidal ideation may be subject to underreporting because of mental-health stigma. Finally, the low prevalence of suicidal ideation limited the power to detect associations beyond depression.

Despite these limitations, the study provides important insights into mental-health differences between PrEP users and non-users in a key population setting in northern Thailand. The findings support integrating mental-health screening and support within HIV-prevention services, given the substantial burden of depressive symptoms among MSM and TGW. Routine assessment of depression, stress, and suicidality, together with referral pathways and brief psychosocial interventions, may strengthen both mental health care and HIV prevention efforts.

Our finding of a substantial burden of depressive symptoms among MSM and TGW is consistent with global evidence showing elevated depression in this population. A 2022 meta-analysis estimated that depression among MSM was nearly three times more common than in the general male population, highlighting the persistent mental-health inequality faced by this group ( Nouri et al., 2022). Longitudinal research among MSM without HIV in China has also shown that anxiety and depression are highly prevalent and remain elevated over time, underscoring the chronic nature of psychological distress in MSM communities ( Wu et al., 2022). Among transgender women, high levels of depression have been reported in several Asian settings. A study of trans women in Bangkok found that more than half met criteria for depression, and low perceived social support was strongly associated with depressive symptoms, which is similar to the pattern of stress and support observed in our data ( Tantirattanakulchai & Hounnaklang, 2021).

The lower depression scores among PrEP users in our study are in line with emerging literature on the mental-health dimensions of PrEP. A 2023 study examining PrEP, anxiety, and depression among MSM found that mental-health concerns such as depression and anxiety were closely related to PrEP use patterns and impacted broader quality of life and sexual satisfaction, suggesting that PrEP is embedded within a wider psychosocial context rather than being a purely biomedical intervention ( Reiriz et al., 2023). More recently, a 2024 study of Chinese MSM initiating PrEP showed distinct trajectories of anxiety and depression during PrEP use and concluded that mental-health support should be integrated into PrEP programs to sustain adherence and well-being ( Chen et al., 2024). Together, these findings support our interpretation that engagement in PrEP services, particularly in community-based settings, may be associated with more favourable mental-health profiles, while also highlighting that PrEP users remain vulnerable to stress and require ongoing psychosocial support.

The patterns observed in this study mirror broader Southeast Asian evidence showing substantial mental-health challenges among LGBTQ+ communities. A recent paper on Thai TGW highlighted that depression prevalence was many times higher than in the general population and that perceived low social support dramatically increased the odds of depression, reinforcing the importance of social and structural determinants (Tantirattanakulchai & Hounnaklang, 2021). Regional work has similarly emphasized that stigma, discrimination, and limited access to affirming mental-health care remain key drivers of poor outcomes, underscoring the need for integrated HIV and mental-health services tailored to MSM and TGW ( Chaovanalikit et al., 2022).

Our findings from Chiang Mai are consistent with these national patterns and suggest that MSM and TGW engaging in HIV prevention services still face substantial mental-health needs that are not routinely addressed in clinical care. A strength of this work is the use of validated measures and multivariable models to explore multiple psychosocial outcomes within a key population.

## Conclusion

These findings support the integration of mental-health screening and support within HIV-prevention settings in Thailand. Community-based PrEP programs offer a valuable platform for routine mental-health screening, early identification of depression, and referral of individuals requiring additional care. However, the observed association between PrEP use and lower depression severity should be interpreted with caution, as the cross-sectional design does not allow conclusions about causality or temporal relationships. This association may reflect differences in healthcare engagement, access to supportive services, social connectedness, or other unmeasured factors rather than a direct effect of PrEP use itself. Strengthening mental-health services within HIV-prevention programs may help address unmet mental-health needs among MSM and TGW. Future research should employ longitudinal designs to clarify the temporal relationships between PrEP engagement, psychosocial stressors, and mental-health trajectories. Studies conducted across diverse regions of Thailand are also needed to inform more inclusive and comprehensive mental-health strategies for key populations.

## Ethical considerations

The study protocol was reviewed and approved by the Institutional Review Board of Chiang Mai University (certificate no.41/67). Written informed consent was obtained from all participants before data collection.

## Data Availability

The data that support the findings of this study can be accessed from Zenodo repository. Dataset_depression and suicidal ideation in Thai MSM [Data set]. Zenodo. https://doi.org/10.5281/zenodo.20061760 ( Lynn, K. S., 2026).
