# Depression and Suicidal Ideation Among Men who have sex with men and Transgender Women in Chiang Mai, Thailand

**DOI:** 10.12688/f1000research.181753.3

**Published:** 2026-08-11

**Authors:** Kyaw Sein Lynn, Linda Aurpibul, Nuntisa Chotirosniramit, Natthapol Kosashunhanan, Kriengkrai Srithanaviboonchai, Awirut Oon-arom

**Affiliations:** 1 School of Health Sciences Research, Research Institute for Health Sciences, Chiang Mai University, Chiang Mai, Chiang Mai, 50200, Thailand; 2 Research Institute for Health Sciences, Chiang Mai University, Chiangmai, CHIANG MAI, 50200, Thailand; 3 Psychiatry, Chiang Mai University Faculty of Medicine, Chiang Mai, Chiang Mai, 50200, Thailand

**Keywords:** Depression, Suicidal Ideation, PrEP users, men who have sex with men, transgender women

## Abstract

**Background:**

Depression and suicidal ideation are common among men who have sex with men (MSM) and transgender women (TGW). Pre-exposure prophylaxis (PrEP) has become an important component of HIV prevention, but its relationship with mental health remains incompletely understood. This study compared depression and suicidal ideation/risk between PrEP users and non-users among MSM and TGW attending HIV prevention services, and identified factors associated with depression.

**Methods:**

A cross-sectional study was conducted among 150 MSM and TGW (75 PrEP users and 75 non-users) recruited from two HIV prevention clinics in Chiang Mai, Thailand Depressive symptoms were assessed using the Patient Health Questionnaire-9 (PHQ-9), and suicidal ideation/risk was assessed using the Columbia-Suicide Severity Rating Scale (C-SSRS). Group differences were compared using χ ^2^ tests, Fisher’s exact test, or independent-samples *t*-tests as appropriate. Multivariable linear regression was performed to identify factors associated with depression.

**Results:**

PrEP users had significantly lower mean depression scores than non-users (3.80±3.46 vs. 5.96±3.76; mean difference 2.16, 95% CI: 0.99–3.33; *p* <0.001). Suicidal ideation/risk was reported by 5 (6.7%) PrEP users and three (4.0%) non-users, with no significant difference between groups (Fisher’s exact test, *p* = 0.719). In the adjusted linear regression model, PrEP use was independently associated with lower depression scores (B = −1.77, 95% CI: −3.07 to −0.47; *p* = 0.008), whereas experience of discrimination was independently associated with higher depression scores (B = −1.32, 95% CI: −2.59 to −0.06; *p* = 0.040).

**Conclusions:**

Among MSM and TGW attending HIV prevention services in Chiang Mai, PrEP use was independently associated with lower depression scores, whereas suicidal ideation/risk did not differ significantly between PrEP users and non-users. Experience of discrimination was associated with higher depression scores. Although causal inferences cannot be made, the findings highlight the importance of considering mental health alongside HIV prevention services for key populations.

## Introduction

Men who have sex with men (MSM) and transgender women (TGW) are HIV key populations, classified respectively by sexual behavior and gender identity rather than by sexual orientation. Alongside these behaviorally and demographically defined groups, sexual and gender minority populations more broadly, encompassing lesbian, gay, bisexual, transgender, queer, and questioning (LGBTQ+) identities, experience a substantially higher burden of mental health problems than the general population. A recent systematic review and meta-analysis estimated that the pooled global prevalence of depression among MSM is about 35%, which is roughly three times more common than in the general male population ( Nouri et al., 2022). Recent systematic reviews further indicate that elevated rates of depression and suicidal ideation persist across diverse settings despite legal and policy advances, largely because stigma, discrimination, minority stress, and other interacting psychosocial factors continue to adversely affect mental health ( Operario et al., 2022; Pellicane & Ciesla, 2022). This evidence highlights the need for context-specific research to better understand factors associated with mental health among MSM and transgender women in Thailand.

In Thailand, emerging evidence indicates that LGBTQ+ populations continue to experience substantial mental-health challenges. Recent studies have highlighted high levels of psychological distress among sexual and gender minority individuals, with a survey of Thai medical students reporting notably elevated rates of depressive and anxiety symptoms among those identifying as LGBTQ+ ( Vadhanavikkit et al., 2025). Research among TGW in Bangkok has also documented a high prevalence of depressive symptoms, with low perceived social support identified as a significant contributing factor ( Tantirattanakulchai & Hounnaklang, 2021). These findings reflect broader patterns observed across Southeast Asia, where stigma, discrimination, and social marginalization continue to shape mental-health disparities among MSM and TGW. Despite these concerns, systematic data on mental-health outcomes among key populations in northern Thailand remain limited, underscoring the need for focused research in this context.

Pre-exposure prophylaxis (PrEP) has become a central component of HIV prevention for MSM and TGW, offering highly effective protection when taken consistently ( Davies et al., 2016; Naswa & Marfatia, 2011; Turner et al., 2018). Beyond its biomedical benefits, the relationship between PrEP use and mental health is complex. Mental health concerns such as depression and anxiety are common among individuals at heightened risk for HIV and may hinder both PrEP uptake and adherence ( Hennessy et al., 2023; Miller et al., 2021). At the same time, PrEP-related stigma—arising from misconceptions within and outside LGBTQ+ communities—can influence willingness to initiate PrEP and contribute to psychological distress. Conversely, engagement with PrEP services may provide opportunities for social support and improved emotional well-being through regular contact with affirming healthcare settings. These intersecting dynamics highlight the importance of understanding mental-health profiles among PrEP users and non-users in Thailand ( Protiere et al., 2023; Sarah M. Wood, 2021; Sun et al., 2019; Yigit et al., 2022). In Thailand, PrEP delivery has expanded through the Key Population-Led Health Services (KPLHS) model, including in Chiang Mai, where community-based organizations provide HIV prevention services tailored to MSM, TGW, and other key populations. These settings aim to increase accessibility and reduce stigma related to HIV prevention. Although KPLHS clinics often offer more affirming environments compared with conventional healthcare facilities, stigma and limited service uptake remain challenges in several contexts.

However, existing Thai research has focused largely on PrEP-related stigma, provider attitudes, and implementation issues ( Chautrakarn et al., 2022; Vannakit et al., 2020), with limited attention to mental-health profiles of clients who used or did not use PrEP. Therefore, this study aimed to assess depression and suicidal ideation in MSM and TGW who were attending HIV prevention services in Chiang Mai, Thailand. We also looked for differences in mental health outcomes between PrEP users and non-users and identified potential associated factors. Understanding these patterns may support the development of integrated HIV prevention and mental-health services tailored to the needs of key populations.

## Materials and methods

### Study setting and participants

PrEP was first introduced in Thailand in 2016 and has subsequently become an integral component of the national HIV prevention programme ( Phanuphak et al., 2018). PrEP is available through public hospitals, private hospitals, and community-led health services for individuals at risk of HIV acquisition, including key populations. This cross-sectional study was conducted in Chiang Mai, Northern Thailand in 2025. At the time of this study, PrEP services are available at more than 20 government hospitals, more than 10 private hospitals, and several community-led clinics ( Data Center, Chiang Mai Provincial Health office, 2025). Among these, **PIMAN Clinic**, a university-affiliated research clinic, and **MPLUS Polyclinic**, a Key Population-Led Health Service (KPLHS) operated by a community-based organization, were selected because they are the principal PrEP service providers for MSM and TGW in Chiang Mai. Located approximately 7 km apart, these clinics serve approximately 850 active PrEP clients and represent Thailand’s two major PrEP service delivery models: healthcare facility-based and community-led services. Participants in the two clinics represented the key population residing in urban area and had comparable demographics; some of them alternated between the two clinics.

To align with the programmatic eligibility criteria for PrEP services, the study population comprised individuals assigned male at birth and transgender women who reported sexual activity with men. The study was designed to compare two predefined exposure groups: participants who had used PrEP continuously for at least six months and participants who had never used PrEP. Individuals who had previously initiated but discontinued PrEP were excluded because they did not meet either predefined exposure category.

Participants were recruited after completing their routine clinic services. At both study sites, HIV testing or HIV status verification was routinely performed according to standard clinical protocols before recruitment into HIV prevention services. Consequently, recruitment was restricted to clients eligible for HIV prevention services, and individuals living with HIV were not approached for enrolment.

Recruitment was conducted through a purposive non-probability sampling strategy, aiming for a total sample size of 150 participants. A sample size of 150 participants was selected based on the commonly reported depression prevalence of approximately 35% among MSM ( Nouri et al., 2022). With this sample, the 95% confidence interval around a 35% prevalence estimate would have a margin of error of about ±8%, which was considered adequate for descriptive purposes. The study was therefore considered adequately powered for descriptive analyses and exploratory analyses of associations between PrEP use and mental health outcomes.

Recruitment was conducted between March and May 2025. A total of 150 participants were enrolled, comprising 75 PrEP users and 75 non-users. Although 75 participants were recruited from each clinic, recruitment site was recorded only for PrEP users because this information was not captured on the questionnaire for non-users. Inclusion criteria were: (1) aged ≥20 years; (2) being an individual assigned male at birth who reported sexual activity with men or identifying as a transgender woman; and (3) either a current PrEP user with at least six months of continuous use or an individual who had never used PrEP. Trained research assistants screened clinic appointment logs, approached eligible clients in waiting areas, obtained written informed consent, and administered structured questionnaires in private consultation rooms. The following variables and measures were collected.

### Data collection

• Socio-demographic Factors

Sociodemographic variables included age, self-identified gender identity (male, female, transgender woman, or non-binary), education level, living arrangement, employment status, and monthly income.

Perceived community safety was assessed using the question, “Do you feel safe in your current living community?” Responses were dichotomized as “Always safe” and “Not always safe.” Current stress was assessed using a single-item self-report question: “How would you rate your current stress level?” Responses were dichotomized as “High stress” and “Low/no stress.” LGBTQ+ community involvement was assessed by asking whether participants actively participated in LGBTQ+-related community groups or activities (Yes/No).

Some participants self-identified as female or non-binary; however, they were included because they met the behavioural eligibility criteria for MSM or TGW. This approach is consistent with Thailand’s PrEP programme, which defines key populations primarily according to behavioural risk and gender identity relevant to HIV prevention services rather than identity labels alone.

• Depression

Depression severity was measured using the Patient Health Questionnaire-9 (PHQ-9) ( Kroenke et al., 2001). The PHQ-9 consists of nine items assessing depressive symptoms during the previous two weeks using a four-point Likert scale ranging from 0 (“Not at all”) to 3 (“Nearly every day”). Total scores range from 0 to 27, with higher scores indicating more severe depressive symptoms.

The PHQ-9 total score was used as the primary continuous outcome in all analyses. The Thai version of the PHQ-9 has demonstrated good reliability and validity for screening major depression, with a Cronbach’s alpha of 0.79 yielding 84% sensitivity and 77% specificity ( Lotrakul et al., 2008).

• Suicidal Ideation/Risk

Suicidal ideation and risk were assessed using the Columbia-Suicide Severity Rating Scale (C-SSRS) Screen Version ( Posner et al., 2011). The instrument has demonstrated good convergent and divergent validity with high sensitivity and specificity. A validated Thai version is available through the Columbia Lighthouse Project ( The Columbia Lighthouse Project, 2016) Responses to the six dichotomous items (wish to be dead, suicidal thoughts, method, intent, plan, and suicidal behaviour) were recorded. Participants endorsing one or more items were classified as having suicidal ideation/risk, whereas those endorsing none were classified as having no suicidal ideation/risk.

### Data analysis

All analyses were conducted using Jamovi version 2.7 (The jamovi Project, Sydney, Australia). Data cleaning included verification of data entry, identification of out-of-range values, and correction of entry errors where applicable.

Descriptive statistics were used to summarize participants’ sociodemographic characteristics, psychosocial factors, and mental health outcomes. Categorical variables were presented as frequencies and percentages, whereas continuous variables were summarized using means and standard deviations. Comparisons between PrEP users and non-users were performed using the χ ^2^ test (or Fisher’s exact test when expected cell counts were <5) for categorical variables and the independent-samples *t*-test for continuous variables. The Mann–Whitney *U* test was used when continuous variables were not normally distributed.

To identify factors associated with depression, multivariable linear regression analysis was performed using the continuous PHQ-9 total score as the dependent variable. Independent variables entered into the model were age group, education level, employment status, monthly income, experience of discrimination, family/societal pressure, community safety, current stress level, social support, LGBTQ+ community involvement, and PrEP use. Unstandardized regression coefficients (B), 95% confidence intervals (CIs), and *p*-values were reported. Model assumptions were assessed by examining residual plots for linearity and homoscedasticity, while multicollinearity was evaluated using variance inflation factors (VIFs), with values <2 indicating no evidence of problematic multicollinearity.

Because suicidal ideation/risk occurred infrequently, resulting in too few outcome events for reliable multivariable modelling, logistic regression was not performed. Instead, Fisher’s exact test was used to compare suicidal ideation/risk between PrEP users and non-users.

## Results

### Participant characteristics

A total of 150 participants were included in the analysis, comprising 75 PrEP users and 75 non-users. Sociodemographic and psychosocial characteristics by PrEP use status are presented in Table 1. Of the 150 participants, the majority self-identified as MSM (76.0%), followed by transgender women (16.0%), non-binary individuals (5.3%), and a small proportion biological males who identified themselves as female (2.7%).

**Table 1.** Comparison of sociodemographic and psychosocial characteristics by PrEP use status.

Characteristic	PrEP users (n = 75)	Non-users (n = 75)	p-value
**Age group**			0.031
20–40 years	65 (86.7%)	73 (97.3%)	
After 40 years	10 (13.3%)	2 (2.7%)	
**Education level**			0.541
High/Vocational school or lower	13 (17.3%)	17 (22.7%)	
Undergraduate or higher	62 (82.7%)	58 (77.3%)	
**Living arrangement**			0.252
Living alone	39 (52.0%)	31 (41.3%)	
Living with others	36 (48.0%)	44 (58.7%)	
**Employment status**			0.025
Unemployed/student	13 (17.3%)	26 (34.7%)	
Employed	62 (82.7%)	49 (65.3%)	
**Monthly income**			1.000
Low income (<20,000 THB or none)	48 (64.0%)	49 (65.3%)	
Higher income (>20,000 THB)	27 (36.0%)	26 (34.7%)	
**Experience of discrimination**			0.506
Experienced discrimination	47 (62.7%)	42 (56.0%)	
No discrimination	28 (37.3%)	33 (44.0%)	
**Family/Society pressure**			0.142
High pressure	16 (21.3%)	25 (33.3%)	
Low/No pressure	59 (78.7%)	50 (66.7%)	
**Community safety**			0.048
Always safe	48 (64.0%)	35 (46.7%)	
Not always safe	27 (36.0%)	40 (53.3%)	
**Current stress level**			0.013
High stress	0 (0.0%)	7 (9.3%)	
Low stress	75 (100.0%)	68 (90.7%)	
**Social support**			0.120
Has support	75 (100.0%)	71 (94.7%)	
No support	0 (0.0%)	4 (5.3%)	
**LGBTQ+ community involvement**			0.009
Yes	66 (88.0%)	52 (69.3%)	
No	9 (12.0%)	23 (30.7%)	

PrEP: pre-exposure prophylaxis; THB: Thai baht; LGBTQ: Lesbian, gay, bisexual, transgender, queers plus.

Compared with non-users, PrEP users were more likely to be older than 40 years (13.3% vs. 2.7%, p = 0.031), employed (82.7% vs. 65.3%, p = 0.025), report always feeling safe in their community (64.0% vs. 46.7%, p = 0.048), and actively participate in LGBTQ+ community activities (88.0% vs. 69.3%, p = 0.009). High stress was reported only among non-users (9.3% vs. 0.0%, p = 0.013). No statistically significant differences were observed for education level, living arrangement, monthly income, experience of discrimination, family/societal pressure, or social support (all p > 0.05).

### Depression and suicidal ideation

Mental health outcomes according to PrEP use are presented in Table 2. PrEP users had significantly lower mean depression scores than non-users (3.80 ± 3.46 vs. 5.96 ± 3.76), with a mean difference of 2.16 (95% CI: 0.99–3.33; p < 0.001).

**Table 2.** Mental health outcomes among PrEP users and non-users.

Variable	PrEP users	Non-users	Effect estimate	p-value
Depression score (Mean ± SD)	3.80 ± 3.46	5.96 ± 3.76	Mean difference = 2.16 (95% CI: 0.99–3.33)	<0.001
Suicidal ideation/risk, n (%)	5 (6.7%)	3 (4.0%)	—	0.719

PrEP: pre-exposure prophylaxis; SD: standard deviation; CI: confident interval.

Suicidal ideation/risk was reported by 5 (6.7%) PrEP users and 3 (4.0%) non-users. Fisher’s exact test showed no statistically significant difference between the two groups (p = 0.719).

### Factors associated with depression

A multivariable linear regression model was performed to identify factors associated with depression scores ( Table 3). The multivariable model explained 17.1% of the variance in depression scores (R ^2^ = 0.171; adjusted R ^2^ = 0.098). After adjustment for demographic and psychosocial factors, participants who did not report experiencing discrimination had significantly lower depression scores than those who had experienced discrimination (B = −1.32, 95% CI: −2.59 to −0.06; p = 0.040). PrEP use was independently associated with lower depression scores (B = −1.77, 95% CI: −3.07 to −0.47; p = 0.008). Age group, education level, employment status, monthly income, family/societal pressure, community safety, current stress level, social support, and LGBTQ+ community involvement were not significantly associated with depression scores (all p > 0.05).

**Table 3.** Linear regression for depression severity.

Predictors	B	95% CI	p-value
Age group (40–59 years vs. 20–39 years)	-0.34	-2.27, 1.59	0.730
Age group (≥60 years vs. 20–39 years)	1.16	-6.22, 8.54	0.756
Education level (Undergraduate or higher vs. High/Vocational school or lower)	0.36	-1.17, 1.90	0.640
Employment status (Employed vs. Unemployed/student)	0.10	-1.37, 1.57	0.896
Monthly income (Higher income vs. Low income)	-0.26	-1.56, 1.05	0.699
Experience of discrimination (No discrimination vs. Experienced discrimination)	**-1.32**	**-2.59, -0.06**	**0.040**
Family/Society pressure (Low/No pressure vs. High pressure)	-0.71	-2.12, 0.71	0.325
Community safety (Not always safe vs. Always safe)	0.68	-0.57, 1.93	0.284
Current stress level (High stress vs. Low stress)	2.37	-0.49, 5.23	0.103
Social support (No support vs. Has support)	-0.97	-4.79, 2.85	0.617
LGBTQ+ community involvement (Yes vs. No)	-0.40	-1.93, 1.12	0.603
PrEP use (PrEP user vs. Non-user)	**-1.77**	**-3.07, -0.47**	**0.008**

PrEP: pre-exposure prophylaxis; LGBTQ: Lesbian, gay, bisexual, transgender, queers plus.

Model fit: R ^2^ = 0.171; Adjusted R ^2^ = 0.098.

### Suicidal ideation

Suicidal ideation was reported by 8 of the 150 participants (5.3%). Among PrEP users, 5 participants (6.7%) reported suicidal ideation compared with 3 participants (4.0%) among non-users ( Table 4). Fisher’s exact test showed no statistically significant association between PrEP use and suicidal ideation (p = 0.719).

**Table 4.** Bivariable analysis for suicidal ideation.

PrEP Use	No suicidal ideation	Suicidal ideation	Total
Non-user	72 (96.0%)	3 (4.0%)	75
PrEP user	70 (93.3%)	5 (6.7%)	75

## Discussion

This study compared depression severity and suicidal ideation/risk between PrEP users and non-users among MSM and TGW attending HIV prevention services in Chiang Mai, Thailand. PrEP users had significantly lower depression scores than non-users, whereas suicidal ideation/risk was infrequent and did not differ significantly between the two groups. After adjustment for demographic and psychosocial factors, PrEP use remained independently associated with lower depression scores, while participants who had experienced discrimination reported significantly higher depression scores.

### Depression among MSM and TGW

The substantial burden of depressive symptoms observed in this study is consistent with previous research demonstrating disproportionately high rates of depression among MSM and TGW. A 2022 meta-analysis estimated that depression among MSM was approximately threefold higher than in the general male population, highlighting the persistent mental-health inequality faced by this group ( Nouri et al., 2022). Longitudinal research among MSM without HIV in China has also shown that anxiety and depression are highly prevalent and remain elevated over time, underscoring the chronic nature of psychological distress in MSM communities ( Wu et al., 2022). Among transgender women, high levels of depression have been reported in several Asian settings. A study of trans women in Bangkok found that more than half met criteria for depression, and low perceived social support was strongly associated with depressive symptoms, which is similar to the pattern of stress and support observed in our data ( Tantirattanakulchai & Hounnaklang, 2021).

The lower depression scores among PrEP users in our study are in line with emerging literature on the mental-health dimensions of PrEP. A 2023 study examining PrEP, anxiety, and depression among MSM found that mental-health concerns such as depression and anxiety were closely related to PrEP use patterns and impacted broader quality of life and sexual satisfaction, suggesting that PrEP use occurs within a broader psychosocial context rather than being a purely biomedical intervention ( Reiriz et al., 2023). More recently, a 2024 study of Chinese MSM initiating PrEP showed distinct trajectories of anxiety and depression during PrEP use and concluded that mental-health support should be integrated into PrEP programs to sustain adherence and well-being ( Chen et al., 2024).

In the present study, PrEP use remained independently associated with lower depression scores after adjustment for demographic and psychosocial factors. However, because of the cross-sectional design, these findings should be interpreted as associations rather than evidence of causality. Individuals with better baseline psychosocial functioning may have been more likely to access, initiate, and remain engaged in PrEP services, and reverse causality cannot be excluded. Longitudinal studies are needed to clarify the temporal relationship between PrEP engagement and mental health outcomes.

### Discrimination and depression

Experience of discrimination was independently associated with higher depression scores in the adjusted analysis. This finding is consistent with minority stress theory, which proposes that stigma and discrimination contribute to adverse mental health outcomes among sexual and gender minority populations ( Pellicane & Ciesla, 2022). Persistent experiences of discrimination may increase psychological distress through chronic exposure to social exclusion, prejudice, and reduced access to supportive environments. This finding further emphasizes the importance of addressing discrimination as part of broader strategies to improve mental health among sexual and gender minority populations.

Beyond discrete experiences of discrimination, broader dimensions of stigma are also likely relevant to depression in this population; a composite stigma score collected alongside this study showed a modest but statistically significant correlation with depression score (r = 0.246, p = 0.002), consistent with minority stress theory, and is explored in greater depth in a companion analysis.

### Suicidal ideation

Suicidal ideation/risk was relatively uncommon in this study and did not differ significantly between PrEP users and non-users. Because only eight participants reported suicidal ideation/risk, the study had limited statistical power to detect between-group differences. Nevertheless, suicidal ideation among MSM and TGW remains an important public health concern, and routine screening may still be warranted within HIV prevention services.

### Strength

A major strength of this study was the use of validated Thai versions of the PHQ-9 and C-SSRS to assess mental health outcomes among MSM and TGW. In addition, the study compared PrEP users and non-users recruited from two major HIV prevention service providers in Chiang Mai and adjusted for a range of demographic and psychosocial covariates when examining factors associated with depression.

### Limitations

This study has several limitations. First, its cross-sectional design precludes causal inference, and reverse causality cannot be excluded. Individuals with better baseline psychosocial functioning may have been more likely to access and remain engaged in PrEP services.

Second, the study compared long-term PrEP users (≥6 months) with individuals who had never used PrEP. Participants who discontinued PrEP before six months were excluded, which may have introduced survivor bias.

Third, MSM and TGW were analysed together because the number of TGW participants was insufficient to support reliable subgroup analyses. These populations experience distinct forms of minority stress, and future studies with larger samples should examine them separately.

Fourth, recruitment site could not be adjusted for because recruitment site information was not captured. Residual confounding related to differences between service settings therefore cannot be excluded.

Fifth, a comprehensive 28-item stigma scale (capturing enacted, perceived, and internalized stigma) was also collected in the parent study but was not entered as a covariate in the present multivariable model, as it falls outside this study’s pre-specified focus on demographic and psychosocial correlates of depression. An exploratory bivariate analysis showed a statistically significant, modest correlation between the composite stigma score and depression score (Pearson r = 0.246, p = 0.002; shared variance ≈ 6%), consistent with stigma being related to — but not the dominant driver of — depressive symptoms in this sample, and unlikely to have materially confounded the PrEP–depression association reported here. The full stigma scale, including its subscales, is examined in depth in a separate manuscript currently in preparation.

Finally, suicidal ideation/risk was uncommon (n = 8), limiting statistical power to detect associations.

## Conclusion

Among MSM and TGW attending HIV prevention services in Chiang Mai, PrEP users had significantly lower depression scores than non-users, whereas suicidal ideation/risk did not differ significantly between the two groups. After adjustment for demographic and psychosocial factors, PrEP use was independently associated with lower depression scores, while experience of discrimination was associated with higher depression scores. Although causal relationships cannot be inferred because of the cross-sectional design, these findings suggest that mental health should be considered alongside HIV prevention when planning services for MSM and TGW. Future longitudinal studies are needed to clarify the temporal relationship between PrEP engagement, psychosocial factors, and mental health outcomes.

## Ethical considerations

The study protocol was reviewed and approved by the Institutional Review Board of Chiang Mai University (certificate no.41/67). Written informed consent was obtained from all participants before data collection.

## Data Availability

The data that support the findings of this study can be accessed from Zenodo repository. Dataset_depression and suicidal ideation in Thai MSM [Data set]. Zenodo. https://doi.org/10.5281/zenodo.20061760 ( Lynn, K. S., 2026).
