# Supplementary material for: Depression and Suicidal Ideation Among Men who have sex with men and Transgender Women in Chiang Mai, Thailand
Source: F1000Res. 2026 Sep 15;15:772. Originally published 2026 May 21. [Version 4] doi: 10.12688/f1000research.181753.4 (PMC13583347; doi:10.12688/f1000research.181753.4)
Supplement: Peer review history

# Reviewer report: Reviewer response for version 4

**DOI:** 10.5256/f1000research.209952.r521358

**Published:** 2026-09-16

**Authors:** Rodman Turpin

**Affiliations:** 1 George Mason University, Fairfax, USA

The authors have addressed my concerns. I have no further recommendations.

Is the work clearly and accurately presented and does it cite the current literature?

Yes

If applicable, is the statistical analysis and its interpretation appropriate?

Yes

Are all the source data underlying the results available to ensure full reproducibility?

Yes

Is the study design appropriate and is the work technically sound?

Yes

Are the conclusions drawn adequately supported by the results?

Yes

Are sufficient details of methods and analysis provided to allow replication by others?

Yes

Reviewer Expertise:

Epidemiology, HIV, LGBT+ Health, Racial/ethnic minority health

I confirm that I have read this submission and believe that I have an appropriate level of expertise to confirm that it is of an acceptable scientific standard.

# Reviewer report: Reviewer response for version 3

**DOI:** 10.5256/f1000research.206523.r511243

**Published:** 2026-09-03

**Authors:** Rodman Turpin

**Affiliations:** 1 George Mason University, Fairfax, USA

The authors conducted a study of depression and suicidality among MSM and transgender women in Thailand. Overall the study is well-written, informative, and addresses an important public health issue. My main recommendations are to improve clarity and context.

 1. The second paragraph needs to discuss both interpersonal and structural homophobia and transphobia in more detail as minority stressors.

 2. A brief description of what a "Key Population-Led Health Service (KPLHS)" means will be helpful.

 3. Was there any missing data? If so, how was it handled in analyses?

 4. Additional future research recommendations will be helpful.

 5. Additional policy recommendations will also be helpful.

Is the work clearly and accurately presented and does it cite the current literature?

Yes

If applicable, is the statistical analysis and its interpretation appropriate?

Yes

Are all the source data underlying the results available to ensure full reproducibility?

Yes

Is the study design appropriate and is the work technically sound?

Yes

Are the conclusions drawn adequately supported by the results?

Yes

Are sufficient details of methods and analysis provided to allow replication by others?

Yes

Reviewer Expertise:

Epidemiology, HIV, LGBT+ Health, Racial/ethnic minority health

I confirm that I have read this submission and believe that I have an appropriate level of expertise to confirm that it is of an acceptable scientific standard, however I have significant reservations, as outlined above.

# Response

**Published:** 2026-09-04

**Authors:** Linda Aurpibul

**Affiliations:** Chiang Mai University Research Institute for Health Sciences, Chiang Mai, Chiang Mai, Thailand

**Responses to Reviewer comments**

 The authors conducted a study of depression and suicidality among MSM and transgender women in Thailand. Overall, the study is well-written, informative, and addresses an important public health issue. My main recommendations are to improve clarity and context.

 1. The second paragraph needs to discuss both interpersonal and structural homophobia and transphobia in more detail as minority stressors.

**Response:** We thank the reviewer for this suggestion. We have expanded the first paragraph of the Introduction to explicitly distinguish interpersonal and structural forms of homophobia and transphobia as minority stressors, consistent with minority stress theory.

*“These mental-health disparities arise from stressors operating at multiple levels, commonly distinguished as interpersonal stressors, such as direct experiences of discrimination, harassment, and rejection, and structural stressors, such as systemic bias, policy exclusion, and institutional stigma that shape the environments in which MSM and transgender women live These two levels frequently interact and compound one another, consistent with minority stress theory, which conceptualizes chronic exposure to both forms of stigma as a key driver of excess psychological distress in sexual and gender minority populations.”*

 2. A brief description of what a "Key Population-Led Health Service (KPLHS)" means will be helpful.

**Response:** We thank the reviewer for this suggestion. We have added a brief definition of Key Population-Led Health Services (KPLHS) at first mention in the Introduction to clarify this term for readers unfamiliar with this service delivery model.

*Key Population-Led Health Services (KPLHS) are HIV prevention and care services that are designed, delivered, and often staffed by members of the key populations they serve, such as MSM, transgender women, and other communities at elevated HIV risk, typically through community-based organizations rather than conventional government or private healthcare facilities.*

 3. Was there any missing data? If so, how was it handled in analyses?

**Response:** There were no missing data for any of the variables included in this study, as all participants completed the structured questionnaire in full under the supervision of trained research assistants.

 4. Additional future research recommendations will be helpful.

**Response:** We thank the reviewer for this suggestion. We have added it in the manuscript.

***“Future research recommendations***

*Several directions for future research follow from these findings. Longitudinal studies are needed to clarify the temporal relationship between PrEP engagement and mental health outcomes, and larger studies should examine MSM and transgender women as separate subgroups given the distinct forms of minority stress each group experiences. Further work should also examine broader dimensions of stigma, including enacted, perceived, and internalized stigma, building on the exploratory correlation reported here, alongside qualitative research to clarify the mechanisms underlying the PrEP-mental health association observed in this study.* *”*

 5. Additional policy recommendations will also be helpful.

**Response:** We thank the reviewer for this suggestion. We have added it in the manuscript.

***“Policy recommendations***

*These findings support integrating routine mental health screening, such as the PHQ-9, into PrEP service delivery, alongside provider training in LGBTQ+-affirming care to address the association between discrimination and depression observed in this study. Embedding brief suicide risk assessment within routine HIV prevention services and continuing to expand community-led models such as KPLHS may further help reduce barriers to both HIV prevention and mental health support for MSM and transgender women.* *”*

# Reviewer report: Reviewer response for version 3

**DOI:** 10.5256/f1000research.206523.r509943

**Published:** 2026-08-21

**Authors:** Francesca Vedovo

**Affiliations:** 1 University of Trieste, Trieste, Italy

Based on the summary of revisions and the authors’ responses, the main concerns raised in my previous report appear to have been adequately addressed. I therefore confirm that I am happy to amend my previous status from **Approved with reservations** to **Approved**.

Is the work clearly and accurately presented and does it cite the current literature?

Partly

If applicable, is the statistical analysis and its interpretation appropriate?

Partly

Are all the source data underlying the results available to ensure full reproducibility?

Yes

Is the study design appropriate and is the work technically sound?

Partly

Are the conclusions drawn adequately supported by the results?

Partly

Are sufficient details of methods and analysis provided to allow replication by others?

Partly

Reviewer Expertise:

NA

I confirm that I have read this submission and believe that I have an appropriate level of expertise to confirm that it is of an acceptable scientific standard.

# Reviewer report: Reviewer response for version 2

**DOI:** 10.5256/f1000research.203852.r500143

**Published:** 2026-07-16

**Authors:** Ashok Jammigumpula

**Affiliations:** 1 Manipal Academy of Higher Education, Manipal, Karnataka, India

***General Overview***

*I have carefully reviewed the authors' rebuttal letter alongside the newly submitted Version 2 of the manuscript. While the authors' response letter outlines a comprehensive and satisfactory set of revisions, a direct examination of the Version 2 manuscript reveals that the vast majority of these promised revisions have **not** been implemented in the text.*

*Furthermore, the authors' justification for omitting the 28-item Stigma Scale is methodologically unsound and contradicts the stated aims of their own study. Given the severe discrepancies between the rebuttal letter and the submitted manuscript, alongside the ongoing issues of data transparency, I consider the article **Not Approved** in its current form.*

*Below is a detailed breakdown of the critical ongoing flaws.*

***I. Severe Discrepancies Between Rebuttal Letter and Manuscript***

*The authors provided a rebuttal letter detailing explicit changes made to the text regarding terminology, literature, methodology, and limitations. However, a review of Version 2 confirms these changes were entirely omitted from the submitted file:*

- ***Behavior vs. Identity Conflation:*** *The authors claimed they revised the introduction to distinguish between behavioral risk (MSM) and gender identity. However, the manuscript still reads: "Sexual and gender diversity populations, including men who have sex with men (MSM), transgender women (TGW), and other Lesbian, Gay, Bisexual, Transgender, Queer or Questioning...".*
- ***Outdated Literature and Regional Generalizations:*** *The authors claimed to have removed pre-2018 country-specific examples and unsubstantiated regional generalizations. This is incorrect. The text still cites Tomori et al., 2016. The manuscript also still claims the findings "reflect broader patterns observed across Southeast Asia".*
- ***Outdated Terminology:*** *The authors stated they updated pathologizing terminology to "individuals assigned male at birth (AMAB)". The text remains unchanged, continuing to refer to "biological males or transgender women" in the Methods.*
- ***Contradictory Outcome Definitions:*** *The authors promised they clarified the C-SSRS as a binary variable and utilized a Fisher's exact test. However, the Methods section still claims the C-SSRS was summarized into a "single multi-categorical variable". Additionally, the results report a "univariable binary logistic regression," not the promised Fisher's exact test.*
- ***Omission of Promised Limitations (Survivor Bias, HIV Status, Site Clustering):*** *The authors claimed they added specific paragraphs acknowledging survivor bias (excluding early PrEP dropouts), unmeasured site-level clustering, and routine HIV clinical screening protocols to the Methods and Limitations sections. The Methods and Limitations sections lack any mention of these critical clarifications.*
- ***"Double Negative" Drafting Error:*** *The authors stated they corrected the confusing sentence regarding PrEP and depression scores. Instead, the text still reads: "PrEP use was independently negatively associated with lower depression scores (B = -0.231, p = 0.025)". This contradicts both grammatical logic and their own updated Table 3.*

***II. Discrepancies in Statistical Reporting (Table 3)***

1. *The multivariable linear regression model presented in the authors' rebuttal letter differs entirely from the model published in the revised manuscript.*

- *The Table 3 provided in the rebuttal letter contains **12 variables** (including Income, Experience of Discrimination, Family/Society pressure, and Social support).*
- *The actual Table 3 in the Version 2 manuscript contains only **7 variables** (Age, Education, Frequency of current stress, PrEP Use, Employment status, Perceived community safety, and LGBTQ+ community involvement).*

*Reporting a different statistical model to the reviewers than the one submitted in the manuscript fundamentally compromises the integrity of the peer review process.*

***III. Data Transparency and Selective Reporting (The Stigma Scale)***

*The authors acknowledged the collection of a comprehensive 28-item Stigma Scale but defended its complete omission by stating: "the primary objective of the present study was to compare depression and suicidal ideation/risk between PrEP users and non-users and to identify demographic and psychosocial factors associated with depression."*

*This justification is a fundamental contradiction. **Stigma is universally recognized as one of the most critical** **psychosocial factors** driving mental health disparities among sexual and gender-diverse populations. To explicitly state that the study's objective is to identify "psychosocial factors associated with depression," while simultaneously burying a fully collected 28-item psychometric scale measuring a paramount psychosocial factor, constitutes severe selective reporting.*

*The authors further admitted in their rebuttal that an exploratory analysis revealed a **significant correlation between stigma and depression (p=0.002)**. There is no mention of the omitted 28-item Stigma scale or its correlation with depression anywhere in the Version 2 Discussion. Selectively omitting significant data because it was "not included in the prespecified multivariable model" is unacceptable in rigorous scientific publishing and obscures vital public health nuances.*

***Conclusion***

*Because the submitted Version 2 manuscript fails to implement the essential corrections outlined in the rebuttal letter, relies on inconsistent statistical reporting, and defends the selective omission of highly relevant psychosocial data, this manuscript is **Not Approved** in its current form.*

Is the work clearly and accurately presented and does it cite the current literature?

Partly

If applicable, is the statistical analysis and its interpretation appropriate?

Partly

Are all the source data underlying the results available to ensure full reproducibility?

Partly

Is the study design appropriate and is the work technically sound?

Partly

Are the conclusions drawn adequately supported by the results?

Partly

Are sufficient details of methods and analysis provided to allow replication by others?

No

Reviewer Expertise:

I am a psychiatrist by training with keen interest in the mental health of LGBTQ+ and sexual and gender diverse populations, exploring the unique psychiatric needs and disparities within these communities

I confirm that I have read this submission and believe that I have an appropriate level of expertise to state that I do not consider it to be of an acceptable scientific standard, for reasons outlined above.

# Reviewer report: Reviewer response for version 1

**DOI:** 10.5256/f1000research.200630.r489301

**Published:** 2026-06-26

**Authors:** Ashok Jammigumpula

**Affiliations:** 1 Manipal Academy of Higher Education, Manipal, Karnataka, India

The authors address a vital and clinically relevant public health intersection: mental health disparities among sexual and gender-diverse populations and engagement with HIV prevention services. The use of culturally validated psychometric instruments, such as the Thai versions of the PHQ-9 and the C-SSRS, is a notable methodological strength. However, there are significant conceptual, statistical, and presentational flaws that must be resolved to ensure the scientific validity and rigor of the findings.

 Below is a detailed breakdown of the required revisions.

**I. Conceptual Framework and Literature Review**

- **Behaviour vs. Identity Conflation:** In the introduction, the manuscript groups "men who have sex with men (MSM), transgender women (TGW), and other Lesbian, Gay, Bisexual, Transgender, Queer or Questioning, and other sexual and gender minority identities". This demonstrates an epidemiological conflation. "MSM" is a behavioural categorization used to describe transmission risk networks, whereas LGBTQ+ terms denote psychosocial sexual orientations and gender identities. The text must be revised to clearly distinguish between behavioural risk groups and sexual/gender minority identities.
- **Pooling MSM and TGW Data:** The manuscript evaluates MSM and TGW as a single entity. While public health frameworks frequently group MSM and TGW together as "key populations" due to shared epidemiological HIV vulnerabilities, their social determinants of mental health are vastly different. A transgender woman's experience of minority stress differs fundamentally from the homophobic stigma experienced by cisgender MSM. The authors must either stratify their regression models and descriptive statistics by gender identity (MSM vs. TGW) or explicitly justify in the limitations section why pooling them was necessary (e.g., sample size constraints), acknowledging the specific vulnerabilities this aggregation obscures.
- **Outdated Literature on India:** The authors cite Tomori et al., 2016, to highlight the "elevated risk among these groups" in India. However, the socio-legal landscape in India fundamentally shifted in 2018 with the decriminalization of homosexuality. Citing pre-2018 data fails to reflect the contemporary structural context. The authors must integrate post-2018 literature showing that depression remains high due to syndemic factors despite legislative reforms.
- **Unsubstantiated Regional Generalizations:** The manuscript claims findings "reflect broader patterns observed across Southeast Asia". Southeast Asia is highly heterogeneous regarding LGBTQ+ rights and stigma. The authors must cite specific, recent regional data to substantiate this claim.

**II. Statistical and Methodological Flaws**

- **Mathematical Circularity in Regression Models:** In Table 3, the linear regression model for "Depression Severity" utilizes the "PHQ-9 Diagnosis (Cut-off≥10)" as a predictor. Because the outcome variable (continuous PHQ-9 score) and this predictor (categorized PHQ-9 score) are derived from the exact same scale, this introduces a severe error of mathematical circularity. The model must be completely re-run without this predictor.
- **Severe Overfitting in Suicidal Ideation Analysis:** The study reports a very low frequency of suicidal ideation: 7% among PrEP users and 4% among non-users. In a sample of 150 participants, this equates to roughly 8 total events. Running a multivariable logistic regression model with nine covariates on only 8 events guarantees severe statistical overfitting. A standard statistical rule of thumb requires at least 10 outcome events per predictor variable. This analysis is statistically invalid and should be downgraded to an exploratory bivariate analysis or omitted entirely.
- **Severe Selection and Survivor Bias:** The inclusion criteria define a PrEP user as someone utilizing the medication for ≥6 months, comparing them to never-users. By explicitly excluding early dropouts (individuals who initiated PrEP but stopped within 1-5 months), the researchers introduced a massive survivor bias. Furthermore, the study attempts to correlate long-term PrEP engagement (≥6 months) with the PHQ-9, which strictly measures depressive symptoms over a brief two-week window. This misalignment must be addressed.
- **"Phantom" Variables and Incomplete Models:** In Table 3, "PrEP Adherence" is listed as a predictor. However, this variable is never defined or mentioned in the Methods section, and it is impossible to apply an "adherence" score to the non-user control group. The codebook shows PrEP_Adherence was indeed collected on a 4-point scale (1 = Daily, 2 = Almost daily, 3 = Sometimes, 4 = 2/6 months) and offers no value for "Not Applicable" or "Non-user." Therefore, in a regression model containing *both* PrEP users and non-users, how did the software handle the 75 non-users? If non-users were left blank, the statistical software would have used "listwise deletion," dropping all 75 non-users from the Table 3 regression model entirely. Additionally, the Data Analysis section explicitly claims covariates like monthly income, community safety, and LGBTQ+ involvement were entered into all multivariable models, yet these are omitted from Table 3. The authors must report the full model accurately.
- **Contradictory Outcome Definitions:** In the Methods, the authors state that the C-SSRS responses were summarized into a "single multi-categorical variable". However, in the Data Analysis section, they claim to use a "binary logistic regression model" for suicidal ideation. This inconsistency makes the suicidality analysis unreproducible and must be clarified.
- **Inter-site Demographic Discrepancies:** The study recruited from two vastly different clinical environments: PIMAN Clinic (a research-based university clinic) and MPLUS Polyclinic (a community-based Key Population-Led Health Service). University cohorts typically differ significantly from community-led cohorts in sociodemographic and baseline symptom profiles. The authors must report a comparative analysis of the two recruitment sites to rule out site-level clustering and confounding.
- **Unadjusted Site-Level Clustering:** Standard epidemiological practice dictates that when sampling from distinct clusters, the "site" must be controlled for in the regression models to account for facility-level variance. The failure to control for "Recruitment Site" in Table 3 leaves a massive, unmeasured confounder in the data.
- **Unmeasured Confounding (HIV Status):** The inclusion criteria define non-users simply as individuals who have "never used PrEP". The manuscript fails to specify the HIV status of this group. If individuals living with HIV are included in the non-user cohort, this represents a massive, unmeasured confounding variable, as HIV status independently impacts depression. The authors must clarify the HIV status of the sample and adjust their limitations accordingly.

**III. Data Presentation and Terminology**

- **Misleading Descriptive Statistics in Table 1:** The summary statistics in Table 1 utilize row percentages rather than standard column percentages, completely confusing the interpretation of baseline characteristics. For example, for "High stress", the table displays 0 (0.00%) for PrEP users and 7 (100%) for non-users. This indicates that of the 7 total stressed people, 100% belonged to the non-user group, rather than showing the actual prevalence of high stress within each respective cohort. Table 1 must be entirely recalculated to display column percentages.
- **Causal Language and Reverse Causality:** The PrEP user and non-user groups exhibit significant baseline differences in age, employment, perceived community safety, and stress levels. While the authors acknowledge cross-sectional limitations, the abstract and discussion lean heavily toward causality. The Discussion concludes that "engagement with HIV-prevention services might positively affect the mental health well-being of the key populations". Given the cross-sectional design and the survivor bias mentioned above, this directional leap is unwarranted. Language must be softened to explicitly describe "associations." The lower depression scores might simply reflect reverse causality: individuals with higher baseline psychosocial stability are more capable of accessing and adhering to preventative healthcare.  
- **"Double Negative" Drafting Error:** In the Results section (Factors associated with depression), the authors state that "PrEP use was independently negatively associated with lower depression scores". Mathematically, a negative association with a *lower* score implies a *higher* score. This phrasing is confusing and contradictory. It should be corrected for precision to read "associated with lower depression scores" or "negatively associated with depression severity."
- **Outdated Terminology:** The manuscript describes some participants as "biological males who identified themselves as female". This phrasing is outdated and pathologizing. It must be revised to standard, affirmative terminology, such as "individuals assigned male at birth (AMAB)" or "transgender women."

**IV. Data Transparency and Selective Reporting**

- **The Omitted Stigma Scale: **The study's codebook clearly outlines a comprehensive 28-item Stigma questionnaire that culminates in a continuous Stigma_Score ranging from 0 to 112. However, this entire psychometric scale is completely absent from the manuscript. Omitting a fully collected 28-item scale, especially one directly relevant to the mental health and systemic stress of key populations, is a severe case of selective reporting. The authors must disclose this data or explicitly justify its exclusion (e.g., did the stigma score highly correlate with depression, thereby weakening the primary association with PrEP use?).

Is the work clearly and accurately presented and does it cite the current literature?

Partly

If applicable, is the statistical analysis and its interpretation appropriate?

Partly

Are all the source data underlying the results available to ensure full reproducibility?

Partly

Is the study design appropriate and is the work technically sound?

Partly

Are the conclusions drawn adequately supported by the results?

Partly

Are sufficient details of methods and analysis provided to allow replication by others?

No

Reviewer Expertise:

I am a psychiatrist by training with keen interest in the mental health of LGBTQ+ and sexual and gender diverse populations, exploring the unique psychiatric needs and disparities within these communities

I confirm that I have read this submission and believe that I have an appropriate level of expertise to state that I do not consider it to be of an acceptable scientific standard, for reasons outlined above.

# Response

**Published:** 2026-07-04

**Authors:** Linda Aurpibul

**Affiliations:** Chiang Mai University Research Institute for Health Sciences, Chiang Mai, Chiang Mai, Thailand

Jul 4 ^th^, 2026

 Dear reviewer,

 Thank you for all of your comments. Please find our responses below.

 The authors address a vital and clinically relevant public health intersection: mental health disparities among sexual and gender-diverse populations and engagement with HIV prevention services. The use of culturally validated psychometric instruments, such as the Thai versions of the PHQ-9 and the C-SSRS, is a notable methodological strength. However, there are significant conceptual, statistical, and presentational flaws that must be resolved to ensure the scientific validity and rigor of the findings.

 Below is a detailed breakdown of the required revisions.

**I. Conceptual Framework and Literature Review**

- **Behaviour vs. Identity Conflation:** In the introduction, the manuscript groups "men who have sex with men (MSM), transgender women (TGW), and other Lesbian, Gay, Bisexual, Transgender, Queer or Questioning, and other sexual and gender minority identities". This demonstrates an epidemiological conflation. "MSM" is a behavioural categorization used to describe transmission risk networks, whereas LGBTQ+ terms denote psychosocial sexual orientations and gender identities. The text must be revised to clearly distinguish between behavioural risk groups and sexual/gender minority identities.

**Response: **Thank you for pointing out this important issue. We have revised the text in the 1 ^st^ paragraph of the introduction to make it clear, as follows.

***“*** *Men who have sex with men (MSM) and transgender women (TGW) are HIV key populations, classified respectively by sexual behavior and gender identity rather than by sexual orientation. Alongside these behaviorally and demographically defined groups, sexual and gender minority populations more broadly, encompassing lesbian, gay, bisexual, transgender, queer, and questioning (LGBTQ+) identities, experience a substantially higher burden of mental health problems than the general population.”*

- **Pooling MSM and TGW Data:** The manuscript evaluates MSM and TGW as a single entity. While public health frameworks frequently group MSM and TGW together as "key populations" due to shared epidemiological HIV vulnerabilities, their social determinants of mental health are vastly different. A transgender woman's experience of minority stress differs fundamentally from the homophobic stigma experienced by cisgender MSM. *The authors must either stratify their regression models and descriptive statistics by gender identity (MSM vs. TGW) or explicitly justify in the limitations section why pooling them was necessary (e.g., sample size constraints), acknowledging the specific vulnerabilities this aggregation obscures.*

**Response: **We absolutely agree with the reviewer. Although MSM and TGW were analyzed together as key populations, we acknowledge that these groups experience different forms of minority stress and stigma. Stratified analyses were not performed because the sample size of TGW participants (24(16%)) was insufficient to support reliable subgroup regression models. Future studies with larger TGW samples should examine these populations separately. This issue is addressed in the study limitation at the paragraph before the last of the revised manuscript as follows.

*“Third, MSM and TGW were analysed together because the number of TGW participants was insufficient to support reliable subgroup analyses. These populations experience distinct forms of minority stress, and future studies with larger samples should examine them separately.”*

- **Outdated Literature on India:** The authors cite Tomori et al., 2016, to highlight the "elevated risk among these groups" in India. However, the socio-legal landscape in India fundamentally shifted in 2018 with the decriminalization of homosexuality. Citing pre-2018 data fails to reflect the contemporary structural context. The authors *must integrate post-2018 literature showing that depression remains high due to syndemic factors despite legislative reforms.*

**Response: **We thank the reviewer for this important observation. We have revised the Introduction by removing the pre-2018 country-specific examples and replacing them with *recent systematic review evidence (2022).* The revised text now cites contemporary literature demonstrating that depression and suicidal ideation remain disproportionately common among MSM and transgender women despite legal and policy advances, largely because of persistent stigma, discrimination, and minority stress. The revised text is in the 1 ^st^ paragraph read as follows.

**“** *Recent systematic reviews further indicate that elevated rates of depression and suicidal ideation persist across diverse settings despite legal and policy advances, largely because stigma, discrimination, minority stress, and other interacting psychosocial factors continue to adversely affect mental health* ( Operario et al., 2022; Pellicane & Ciesla, 2022).”

- **Unsubstantiated Regional Generalizations:** The manuscript claims findings "reflect broader patterns observed across Southeast Asia". Southeast Asia is highly heterogeneous regarding LGBTQ+ rights and stigma. The authors must cite specific, recent regional data to substantiate this claim.

**Response: **We thank the reviewer for this important and insightful comment. We agree that Southeast Asia is highly heterogeneous in terms of sociocultural norms, legal frameworks, and levels of stigma toward sexual and gender minority populations. In light of this, we have revised the manuscript to remove the generalized statement referring to “broader patterns observed across Southeast Asia,” as it was not sufficiently supported by specific regional evidence.

 The revised paragraph now focuses more precisely on evidence from Thailand, particularly studies conducted in Bangkok and other local settings, to ensure contextual accuracy and relevance to the study population. This modification improves the conceptual clarity and avoids overgeneralization beyond the available empirical evidence. It is read as follows.

*“In Thailand, emerging evidence indicates that LGBTQ+ populations continue to experience substantial mental-health challenges. Recent studies have highlighted high levels of psychological distress among sexual and gender minority individuals, with a survey of Thai medical students reporting notably elevated rates of depressive and anxiety symptoms among those identifying as LGBTQ+ (Vadhanavikkit et al., 2025). Research among TGW in Bangkok has also documented a high prevalence of depressive symptoms, with low perceived social support identified as a significant contributing factor (Tantirattanakulchai & Hounnaklang, 2021). Despite these concerns, systematic data on mental-health outcomes among key populations in northern Thailand remain limited, underscoring the need for focused research in this context.”*

**II. Statistical and Methodological Flaws**

- **Mathematical Circularity in Regression Models:** In Table 3, the linear regression model for "Depression Severity" utilizes the "PHQ-9 Diagnosis (Cut-off≥10)" as a predictor. Because the outcome variable (continuous PHQ-9 score) and this predictor (categorized PHQ-9 score) are derived from the exact same scale, this introduces a severe error of mathematical circularity. The model must be completely re-run without this predictor.

**Response: **We agree that including the dichotomized PHQ-9 diagnosis as a predictor of the continuous PHQ-9 score introduced mathematical circularity because both variables were derived from the same instrument. Accordingly, we removed PHQ-9 diagnosis from the regression model and re-ran the multivariable analysis using only independent demographic, psychosocial, and PrEP-related variables. Table 3 and the corresponding Results have been revised.

**Predictors**

**B**

**95% CI**

**p-value**

 Age group (40–59 years vs. 20–39 years)

 -0.34

 -2.27, 1.59

 0.730

 Age group (≥60 years vs. 20–39 years)

 1.16

 -6.22, 8.54

 0.756

 Education level

 (Undergraduate or higher vs. High/Vocational school or lower)

 0.36

 -1.17, 1.90

 0.640

 Employment status (Employed vs. Unemployed/student)

 0.10

 -1.37, 1.57

 0.896

 Monthly income (Higher income vs. Low income)

 -0.26

 -1.56, 1.05

 0.699

 Experience of discrimination

 (No discrimination vs. Experienced discrimination)

**-1.32**

**-2.59, -0.06**

**0.040**

 Family/Society pressure (Low/No pressure vs. High pressure)

 -0.71

 -2.12, 0.71

 0.325

 Community safety (Not always safe vs. Always safe)

 0.68

 -0.57, 1.93

 0.284

 Current stress level (High stress vs. Low stress)

 2.37

 -0.49, 5.23

 0.103

 Social support (No support vs. Has support)

 -0.97

 -4.79, 2.85

 0.617

 LGBTQ+ community involvement (Yes vs. No)

 -0.40

 -1.93, 1.12

 0.603

 PrEP use (PrEP user vs. Non-user)

**-1.77**

**-3.07, -0.47**

**0.008**

  

- **Severe Overfitting in Suicidal Ideation Analysis:** The study reports a very low frequency of suicidal ideation: 7% among PrEP users and 4% among non-users. In a sample of 150 participants, this equates to roughly 8 total events. Running a multivariable logistic regression model with nine covariates on only 8 events guarantees severe statistical overfitting. *A standard statistical rule of thumb requires at least 10 outcome events per predictor variable. This analysis is statistically invalid and should be downgraded to an exploratory bivariate analysis or omitted entirely.*

**Response: **We thank the reviewer for this important observation. We agree that the number of suicidal ideation events (n = 8) was insufficient to support a multivariable logistic regression model without substantial risk of overfitting. In response, we have removed the logistic regression analysis and replaced it with an exploratory bivariate analysis using Fisher's exact test, which is more appropriate for sparse binary outcome data. The Results and Methods sections have been revised accordingly.

 The revised text in the last paragraph of the result section is now read as follows.

***“*** *Suicidal ideation was reported by 8 of the 150 participants (5.3%). Among PrEP users, 5 participants (6.7%) reported suicidal ideation compared with 3 participants (4.0%) among non-users. Fisher's exact test showed no statistically significant association between PrEP use and suicidal ideation (p = 0.719).”*

*Table 4 Bivariable analysis for suicidal ideation*

***PrEP Use***

***No suicidal ideation***

***Suicidal ideation***

***Total***

*Non-user*

*72 (96.0%)*

*3 (4.0%)*

*75*

*PrEP user*

*70 (93.3%)*

*5 (6.7%)*

*75*

**Severe Selection and Survivor Bias:** The inclusion criteria define a PrEP user as someone utilizing the medication for ≥6 months, comparing them to never-users. By explicitly excluding early dropouts (individuals who initiated PrEP but stopped within 1-5 months), the researchers introduced a massive survivor bias. Furthermore, the study attempts to correlate long-term PrEP engagement (≥6 months) with the PHQ-9, which strictly measures depressive symptoms over a brief two-week window. This misalignment must be addressed.

**Response: **We appreciate the reviewer's comment. Our intention was to compare two clearly defined groups—participants who had used PrEP continuously for at least six months and participants who had never used PrEP. Individuals who had previously initiated but discontinued PrEP were therefore outside the predefined comparison groups. We acknowledge that this design may introduce survivor bias because long-term PrEP users may differ systematically from those who discontinue treatment. We have clarified the study design in the Methods and added this limitation to the Discussion.

 In the method section:

*“To align with the programmatic eligibility criteria for PrEP services, the study population comprised individuals assigned male at birth and transgender women who reported sexual activity with men. The study was designed to compare two predefined exposure groups: participants who had used PrEP continuously for at least six months and participants who had never used PrEP. Individuals who had previously initiated but discontinued PrEP were excluded because they did not meet either predefined exposure category.“*

 In the limitation section:

***“*** *Second, the study compared long-term PrEP users (≥6 months) with individuals who had never used PrEP. Participants who discontinued PrEP before six months were excluded, which may have introduced survivor bias. **”***

**"Phantom" Variables and Incomplete Models:** In Table 3, "PrEP Adherence" is listed as a predictor. However, this variable is never defined or mentioned in the Methods section, and it is impossible to apply an "adherence" score to the non-user control group. The codebook shows PrEP_Adherence was indeed collected on a 4-point scale (1 = Daily, 2 = Almost daily, 3 = Sometimes, 4 = 2/6 months) and offers no value for "Not Applicable" or "Non-user." Therefore, in a regression model containing *both* PrEP users and non-users, how did the software handle the 75 non-users? If non-users were left blank, the statistical software would have used "listwise deletion," dropping all 75 non-users from the Table 3 regression model entirely. Additionally, the Data Analysis section explicitly claims covariates like monthly income, community safety, and LGBTQ+ involvement were entered into all multivariable models, yet these are omitted from Table 3. The authors must report the full model accurately.

**Response:** We thank the reviewer for identifying this inconsistency. PrEP adherence was measured only among current PrEP users and was therefore not applicable to non-users. Including this variable in the combined regression model was inappropriate and could have resulted in listwise deletion of participants with missing adherence data. We have removed PrEP adherence from the multivariable model and revised the Methods, Results, and Table 3 accordingly. The final model now includes only variables measured consistently across all participants.

**The text and Table 3 in the Result section are noe read as follows.**

*“PrEP use was independently associated with lower depression scores (B = −1.77, 95% CI: −3.07 to −0.47; p = 0.008). Age group, education level, employment status, monthly income, family/societal pressure, community safety, current stress level, social support, and LGBTQ+ community involvement were not significantly associated with depression scores (all p > 0.05).”*

*Table 3. Linear Regression for Depression Severity.*

***Predictors***

***B***

***95% CI***

***p-value***

*Age group (40–59 years vs. 20–39 years)*

*-0.34*

*-2.27, 1.59*

*0.730*

*Age group (≥60 years vs. 20–39 years)*

*1.16*

*-6.22, 8.54*

*0.756*

*Education level*

*(Undergraduate or higher vs. High/Vocational school or lower)*

*0.36*

*-1.17, 1.90*

*0.640*

*Employment status (Employed vs. Unemployed/student)*

*0.10*

*-1.37, 1.57*

*0.896*

*Monthly income (Higher income vs. Low income)*

*-0.26*

*-1.56, 1.05*

*0.699*

*Experience of discrimination*

*(No discrimination vs. Experienced discrimination)*

***-1.32***

***-2.59, -0.06***

***0.040***

*Family/Society pressure (Low/No pressure vs. High pressure)*

*-0.71*

*-2.12, 0.71*

*0.325*

*Community safety (Not always safe vs. Always safe)*

*0.68*

*-0.57, 1.93*

*0.284*

*Current stress level (High stress vs. Low stress)*

*2.37*

*-0.49, 5.23*

*0.103*

*Social support (No support vs. Has support)*

*-0.97*

*-4.79, 2.85*

*0.617*

*LGBTQ+ community involvement (Yes vs. No)*

*-0.40*

*-1.93, 1.12*

*0.603*

*PrEP use (PrEP user vs. Non-user)*

***-1.77***

***-3.07, -0.47***

***0.008***

  

- **Contradictory Outcome Definitions:** In the Methods, the authors state that the C-SSRS responses were summarized into a "single multi-categorical variable". However, in the Data Analysis section, they claim to use a "binary logistic regression model" for suicidal ideation. This inconsistency makes the suicidality analysis unreproducible and must be clarified.

**Response: **We thank the reviewer for identifying this inconsistency. We agree that the original description of the C-SSRS outcome did not correspond to the reported statistical analysis. The Methods have been revised to clarify that responses to the six C-SSRS items were summarized into a binary variable indicating the presence or absence of suicidal ideation/risk, defined as endorsement of one or more C-SSRS items. Because the number of events was small, the original logistic regression analysis was removed and replaced with Fisher's exact test. The Methods and Results sections have been revised accordingly.

 In the data analysis part of the method section:

*“* *To identify factors associated with depression, multivariable linear regression analysis was performed using the continuous PHQ-9 total score as the dependent variable. Independent variables* *entered into the model were* *age group, education level, employment status, monthly income, experience of discrimination, family/societal pressure, community safety, current stress level, social support, LGBTQ+ community involvement, and PrEP use.* *Unstandardized regression coefficients (B)* *, 95% confidence intervals (CIs), and p-values were reported. Model assumptions were assessed by examining residual plots for linearity and homoscedasticity, while multicollinearity was evaluated using variance inflation factors (VIFs), with values <2 indicating no evidence of problematic multicollinearity.*

*Because suicidal ideation/risk occurred infrequently, resulting in too few outcome events for reliable multivariable modelling, logistic regression was not performed. Instead, Fisher's exact test was used to compare suicidal ideation/risk between PrEP users and non-users.”*

 In the result section:

*“Suicidal ideation was reported by 8 of the 150 participants (5.3%). Among PrEP users, 5 participants (6.7%) reported suicidal ideation compared with 3 participants (4.0%) among non-users. Fisher's exact test showed no statistically significant association between PrEP use and suicidal ideation (p = 0.719).”*

*Table 4 Bivariable analysis for suicidal ideation*

***PrEP Use***

***No suicidal ideation***

***Suicidal ideation***

***Total***

*Non-user*

*72 (96.0%)*

*3 (4.0%)*

*75*

*PrEP user*

*70 (93.3%)*

*5 (6.7%)*

*75*

**Inter-site Demographic Discrepancies:** The study recruited from two vastly different clinical environments: PIMAN Clinic (a research-based university clinic) and MPLUS Polyclinic (a community-based Key Population-Led Health Service). University cohorts typically differ significantly from community-led cohorts in sociodemographic and baseline symptom profiles. The authors must report a comparative analysis of the two recruitment sites to rule out site-level clustering and confounding.

- **Unadjusted Site-Level Clustering:** Standard epidemiological practice dictates that when sampling from distinct clusters, the "site" must be controlled for in the regression models to account for facility-level variance. The failure to control for "Recruitment Site" in Table 3 leaves a massive, unmeasured confounder in the data.

**Response: **We thank the reviewer for the important comments regarding the two study sites. Participants were recruited equally from the two study clinics (75 participants per clinic). We added a sentence in the 1 ^st^ paragraph of the method section to specific their characteristics as follows.

***“*** *Participants in the two clinics represented the key population residing in urban area and had comparable demographics; some of them alternated between the two clinics.”*

 We regret to inform you that the comparative analysis of the two recruitment sites cannot be done as we did not capture this information on the questionnaire. Consequently, recruitment site could not be included as a covariate in the multivariable analyses. We have clarified this in the Methods as mentioned above, and acknowledged it as a limitation in the revised manuscript.

*“Fourth, recruitment site could not be adjusted for because recruitment site information was not captured. Residual confounding related to differences between service settings therefore cannot be excluded.*“

- **Unmeasured Confounding (HIV Status):** The inclusion criteria define non-users simply as individuals who have "never used PrEP". The manuscript fails to specify the HIV status of this group. If individuals living with HIV are included in the non-user cohort, this represents a massive, unmeasured confounding variable, as HIV status independently impacts depression. The authors must clarify the HIV status of the sample and adjust their limitations accordingly.

**Response:** We thank the reviewer for raising this important point. We agree that the recruitment process was insufficiently described in the original manuscript. Participants were recruited after completing their routine clinical services at HIV prevention clinics. At both study sites, HIV testing or HIV status verification is routinely performed according to standard clinical protocols before recruitment into HIV prevention services. Individuals with HIV were therefore not targeted for recruitment, and study participants were recruited from clients eligible for HIV prevention services. We have clarified this process in the Methods section. Accordingly, HIV status was not considered a source of confounding in the present study.

 The clarification is in the 3 ^rd^ paragraph of the method section. It is read as follows.

***“*** *Participants were recruited after completing their routine clinic services. At both study sites, HIV testing or HIV status verification was routinely performed according to standard clinical protocols before recruitment into HIV prevention services. Consequently, recruitment was restricted to clients eligible for HIV prevention services, and individuals living with HIV were not approached for enrolment.”*

**III. Data Presentation and Terminology**

- **Misleading Descriptive Statistics in Table 1:** The summary statistics in Table 1 utilize row percentages rather than standard column percentages, completely confusing the interpretation of baseline characteristics. For example, for "High stress", the table displays 0 (0.00%) for PrEP users and 7 (100%) for non-users. This indicates that of the 7 total stressed people, 100% belonged to the non-user group, rather than showing the actual prevalence of high stress within each respective cohort. Table 1 must be entirely recalculated to display column percentages.

**Response: ** We revised Table 1 as follows.

**Table 1. Comparison of sociodemographic and psychosocial characteristics by PrEP use status**

***Characteristic***

***PrEP Users (n = 75)***

***Non-Users (n = 75)***

***p-value***

***Age group***

*0.031*

*20–40 years*

*65 (86.7%)*

*73 (97.3%)*

*After 40 years*

*10 (13.3%)*

*2 (2.7%)*

***Education level***

*0.541*

*High/Vocational school or lower*

*13 (17.3%)*

*17 (22.7%)*

*Undergraduate or higher*

*62 (82.7%)*

*58 (77.3%)*

***Living arrangement***

*0.252*

*Living alone*

*39 (52.0%)*

*31 (41.3%)*

*Living with others*

*36 (48.0%)*

*44 (58.7%)*

***Employment status***

*0.025*

*Unemployed/student*

*13 (17.3%)*

*26 (34.7%)*

*Employed*

*62 (82.7%)*

*49 (65.3%)*

***Monthly income***

*1.000*

*Low income (<20,000 THB or none)*

*48 (64.0%)*

*49 (65.3%)*

*Higher income (>20,000 THB)*

*27 (36.0%)*

*26 (34.7%)*

***Experience of discrimination***

*0.506*

*Experienced discrimination*

*47 (62.7%)*

*42 (56.0%)*

*No discrimination*

*28 (37.3%)*

*33 (44.0%)*

***Family/Society pressure***

*0.142*

*High pressure*

*16 (21.3%)*

*25 (33.3%)*

*Low/No pressure*

*59 (78.7%)*

*50 (66.7%)*

***Community safety***

*0.048*

*Always safe*

*48 (64.0%)*

*35 (46.7%)*

*Not always safe*

*27 (36.0%)*

*40 (53.3%)*

***Current stress level***

*0.013*

*High stress*

*0 (0.0%)*

*7 (9.3%)*

*Low stress*

*75 (100.0%)*

*68 (90.7%)*

***Social support***

*0.120*

*Has support*

*75 (100.0%)*

*71 (94.7%)*

*No support*

*0 (0.0%)*

*4 (5.3%)*

***LGBTQ+ community involvement***

*0.009*

*Yes*

*66 (88.0%)*

*52 (69.3%)*

*No*

*9 (12.0%)*

*23 (30.7%)*

**Causal Language and Reverse Causality:** The PrEP user and non-user groups exhibit significant baseline differences in age, employment, perceived community safety, and stress levels. While the authors acknowledge cross-sectional limitations, the abstract and discussion lean heavily toward causality. The Discussion concludes that "engagement with HIV-prevention services might positively affect the mental health well-being of the key populations". Given the cross-sectional design and the survivor bias mentioned above, this directional leap is unwarranted. Language must be softened to explicitly describe "associations." The lower depression scores might simply reflect reverse causality: individuals with higher baseline psychosocial stability are more capable of accessing and adhering to preventative healthcare.  

**Response: **We thank the reviewer for this thoughtful comment. We agree that the cross-sectional design does not permit causal inference and that reverse causality is a plausible explanation for the observed association. In response, we have revised the Abstract and Discussion to consistently describe PrEP use as being **associated with**, rather than causing, lower depression scores. We have also expanded the Discussion to acknowledge that individuals with better baseline psychosocial functioning may be more likely to access and remain engaged in PrEP services, and that longitudinal studies are required to establish temporality.

 The conclusion of the abstract is now read as follows.

*“* *Among MSM and TGW attending HIV prevention services in Chiang Mai, PrEP use was independently associated with lower depression scores, whereas suicidal ideation/risk did not differ significantly between PrEP users and non-users. Experience of discrimination was associated with higher depression scores. Although causal inferences cannot be made because of the cross-sectional design, the findings highlight the importance of considering mental health alongside HIV prevention services for key populations.”*

 The 1 ^st^ paragraph of the discussion section was revised as follows.

*“This study compared depression severity and suicidal ideation/risk between PrEP users and non-users among MSM and TGW attending HIV prevention services in Chiang Mai, Thailand. PrEP users had significantly lower depression scores than non-users, whereas suicidal ideation/risk was infrequent and did not differ significantly between the two groups. After adjustment for demographic and psychosocial factors, PrEP use remained independently associated with lower depression scores, while participants who had experienced discrimination reported significantly higher depression scores.”*

  

- **"Double Negative" Drafting Error:** In the Results section (Factors associated with depression), the authors state that "PrEP use was independently negatively associated with lower depression scores". Mathematically, a negative association with a *lower* score implies a *higher* score. This phrasing is confusing and contradictory. It should be corrected for precision to read "associated with lower depression scores" or "negatively associated with depression severity."

**Response: **We change that sentence into the correct one regarding new result. The text before Table 3 is now read as follows.

*“PrEP use was independently associated with lower depression scores (B = -1.77, 95% CI: -3.07 to -0.47, p = 0.008).”*

- **Outdated Terminology:** The manuscript describes some participants as "biological males who identified themselves as female". This phrasing is outdated and pathologizing. It must be revised to standard, affirmative terminology, such as "individuals assigned male at birth (AMAB)" or "transgender women."

**Response:** We thank the reviewer for this important comment and the description was changed to “individuals assigned male at birth (AMAB) who identified as female" as suggested in the method section (study population and inclusion criteria), and the results (participant characteristics).

**IV. Data Transparency and Selective Reporting**

- **The Omitted Stigma Scale: **The study's codebook clearly outlines a comprehensive 28-item Stigma questionnaire that culminates in a continuous Stigma Score ranging from 0 to 112. However, this entire psychometric scale is completely absent from the manuscript. Omitting a fully collected 28-item scale, especially one directly relevant to the mental health and systemic stress of key populations, is a severe case of selective reporting. The authors must disclose this data or explicitly justify its exclusion (e.g., did the stigma score highly correlate with depression, thereby weakening the primary association with PrEP use?).

**Response: **We thank the reviewer for this important observation. A comprehensive stigma scale was collected as part of the broader questionnaire. *However, the primary objective of the present study was to compare depression and suicidal ideation/risk between PrEP users and non-users and to identify demographic and psychosocial factors associated with depression. Consequently, the stigma scale was not included in the prespecified multivariable model. Exploratory analysis showed a weak-to-moderate correlation between stigma and depression (Pearson's r = 0.246, p = 0.002), suggesting that omission of this variable is unlikely to have materially altered the primary association between PrEP use and depression. We acknowledge that stigma is an important determinant of mental health and have noted in the Discussion that future studies should further examine its relationship* with depression among MSM and TGW.

# Reviewer report: Reviewer response for version 1

**DOI:** 10.5256/f1000research.200630.r489296

**Published:** 2026-06-01

**Authors:** Francesca Vedovo

**Affiliations:** 1 University of Trieste, Trieste, Italy

This manuscript addresses an important and clinically relevant topic: depression and suicidal ideation among MSM and transgender women attending HIV prevention services in Chiang Mai. The use of validated tools such as PHQ-9 and C-SSRS is a strength, and the integration of mental-health screening into PrEP services is a valuable public-health message. However, several major issues should be addressed before approval.

1. **The causal interpretation should be toned down.**
  The study is cross-sectional, so it cannot establish whether PrEP use leads to lower depression. The authors should consistently state that PrEP use was “associated with” lower depression scores, without implying a protective effect.
2. **PrEP users and non-users differ in several baseline characteristics.**
  The two groups differ in age, employment, perceived community safety, stress level, and LGBTQ+ community involvement. These factors may confound the association between PrEP use and depression and should be discussed more clearly.
3. **The regression model for depression needs revision.**
  Table 3 includes “PHQ-9 diagnosis/cut-off ≥10” as a predictor of PHQ-9 depression score. This is circular, because the predictor is derived from the same scale used as the outcome. This variable should be removed and the model should be repeated.
4. **The analysis of suicidal ideation is likely underpowered.**
  Suicidal ideation was rare in the sample. A multivariable logistic regression with several predictors may therefore be unstable. The authors should simplify this analysis or clearly present it as exploratory.
5. **Tables need careful checking.**
  Some percentages and counts, especially in Table 1, are difficult to interpret and may be inconsistent. The authors should clarify whether percentages are row-based or column-based and correct any discrepancies.
6. **The discussion and conclusion should be more cautious.**
  The findings support mental-health screening within HIV prevention services, but they do not prove that PrEP itself improves mental health. Alternative explanations, such as better healthcare engagement or stronger social support among PrEP users, should be emphasized.

I recommend **major revision**. The topic is relevant and potentially useful, but the statistical analysis and interpretation need important corrections before the manuscript can be approved.

Is the work clearly and accurately presented and does it cite the current literature?

Partly

If applicable, is the statistical analysis and its interpretation appropriate?

Partly

Are all the source data underlying the results available to ensure full reproducibility?

Yes

Is the study design appropriate and is the work technically sound?

Partly

Are the conclusions drawn adequately supported by the results?

Partly

Are sufficient details of methods and analysis provided to allow replication by others?

Partly

Reviewer Expertise:

I am a urologist and andrologist with clinical and research experience in sexual medicine, gender dysphoria, and transgender health. I have been involved in the assessment of sexual function in transgender women and served as the ideator and coordinator of the study group that developed the oMtFSFI questionnaire. My expertise allows me to evaluate this manuscript from the perspective of urology, andrology, sexual health, gender-affirming care, and the methodological assessment of patient-reported outcomes in transgender populations.

I confirm that I have read this submission and believe that I have an appropriate level of expertise to confirm that it is of an acceptable scientific standard, however I have significant reservations, as outlined above.

# Response

**Published:** 2026-06-12

**Authors:** Linda Aurpibul

**Affiliations:** Chiang Mai University Research Institute for Health Sciences, Chiang Mai, Chiang Mai, Thailand

Dear reviewer,

 Thank you for your valuable comments.

 Please find our responses below.

 1. **The causal interpretation should be toned down. **The study is cross-sectional, so it cannot establish whether PrEP use leads to lower depression. The authors should consistently state that PrEP use was “associated with” lower depression scores, without implying a protective effect.

**Response:** Thank you for this comment that we absolutely agree with. The manuscript has been revised and updated. The abstract conclusion is revised as follows.

*“ **Conclusions:** PrEP use was associated with lower depression severity among MSM and TGW in Chiang Mai after adjustment for potential confounders, and frequency of current stress was the strongest associated factor. However, the cross-sectional design precludes causal conclusions, and the association between PrEP use and depression may reflect differences in healthcare engagement or social support rather than a direct effect of PrEP. These findings support integrating routine mental health screening and stress assessment into community-based HIV prevention programs serving MSM and TGW.”*

**2. PrEP users and non-users differ in several baseline characteristics.** The two groups differ in age, employment, perceived community safety, stress level, and LGBTQ+ community involvement. These factors may confound the association between PrEP use and depression and should be discussed more clearly.

**Response:  **This issue was mentioned in the study limitation as flows.

*“…individuals who access and sustain PrEP engagement may already have stronger connections to healthcare services, greater health literacy, and more established relationships with community-based organisations — factors that are independently associated with better mental health outcomes.”*

**3.  The regression model for depression needs revision.**

 Table 3 includes “PHQ-9 diagnosis/cut-off ≥10” as a predictor of PHQ-9 depression score. This is circular, because the predictor is derived from the same scale used as the outcome. This variable should be removed, and the model should be repeated.

**Response:** Thank you for this critical comment. We have removed PHQ-9 diagnosis/cut-off ≥10 and rerun the analysis. The results are presented in Table 3.

**4.The analysis of suicidal ideation is likely underpowered.**

 Suicidal ideation was rare in the sample. A multivariable logistic regression with several predictors may therefore be unstable. The authors should simplify this analysis or clearly present it as exploratory.

**Response:** We have simplified the analysis as presented in Table 4 below. The results on the last paragraph on Page 11 of the manuscript is revised accordingly.

***Factors Associated with Suicidal Ideation***

 An exploratory univariable binary logistic regression was conducted to assess the association between PrEP use and suicidal ideation ( ***Table 4***). PrEP use was not significantly associated with suicidal ideation (OR = 1.714, 95% CI: 0.395–7.446, p = .472). Although the estimated odds of suicidal ideation were higher among PrEP users than non-users, the wide confidence interval and lack of statistical significance indicate insufficient evidence of an association in this sample.

**5. Tables need careful checking.**

 Some percentages and counts, especially in Table 1, are difficult to interpret and may be inconsistent. The authors should clarify whether percentages are row-based or column-based and correct any discrepancies.

**Response:** Thank you for picking this up. We have reviewed and corrected those errors in the revised Tables.

**6. The discussion and conclusion should be more cautious.**

 The findings support mental-health screening within HIV prevention services, but they do not prove that PrEP itself improves mental health. Alternative explanations, such as better healthcare engagement or stronger social support among PrEP users, should be emphasized.

**Response:** We appreciate the reviewer’s thoughtful suggestion and have revised the Discussion and Conclusion accordingly. The text now emphasizes that our findings demonstrate an association rather than a causal effect of PrEP on mental health, and it explicitly discusses alternative explanations such as increased healthcare engagement and stronger social support among PrEP users. The revision can be seen in the uploaded revised manuscript.

 Sincerely,

 Linda
